# Renewal Reward Perspective on Linear Switching Diffusion Systems in Models of Intracellular Transport

**DOI:** 10.1007/s11538-020-00797-w

**Published:** 2020-09-16

**Authors:** Maria-Veronica Ciocanel, John Fricks, Peter R. Kramer, Scott A. McKinley

**Affiliations:** 1 Department of Mathematics and Biology, Duke University, Durham, USA; 2 School of Mathematical and Statistical Sciences, Arizona State University, Tempe, USA; 3 Department of Mathematical Sciences, Rensselaer Polytechnic Institute, Troy, USA; 4 Department of Mathematics, Tulane University, New Orleans, USA

**Keywords:** Renewal reward theory, Intracellular transport, Processive motor transport, 60J20, 60J27, 92B05, 92C37

## Abstract

In many biological systems, the movement of individual agents is characterized having multiple qualitatively distinct behaviors that arise from a variety of biophysical states. For example, in cells the movement of vesicles, organelles, and other intracellular cargo is affected by their binding to and unbinding from cytoskeletal filaments such as microtubules through molecular motor proteins. A typical goal of theoretical or numerical analysis of models of such systems is to investigate effective transport properties and their dependence on model parameters. While the effective velocity of particles undergoing switching diffusion dynamics is often easily characterized in terms of the long-time fraction of time that particles spend in each state, the calculation of the effective diffusivity is more complicated because it cannot be expressed simply in terms of a statistical average of the particle transport state at one moment of time. However, it is common that these systems are regenerative, in the sense that they can be decomposed into independent cycles marked by returns to a base state. Using decompositions of this kind, we calculate effective transport properties by computing the moments of the dynamics within each cycle and then applying renewal reward theory. This method provides a useful alternative large-time analysis to direct homogenization for linear advection–reaction–diffusion partial differential equation models. Moreover, it applies to a general class of semi-Markov processes and certain stochastic differential equations that arise in models of intracellular transport. Applications of the proposed renewal reward framework are illustrated for several case studies such as mRNA transport in developing oocytes and processive cargo movement by teams of molecular motor proteins.

## Introduction

Microscale biological agents frequently change biophysical state, which results in significant changes in their movement behavior. Intracellular cargo, for example, switches among active transport, diffusive transport, and paused states, each resulting from different mechanochemical configurations of the cargo, cytoskeletal filaments, and the molecular motors that bind them (Hancock 2014; Bressloff and Newby 2013). Models for this behavior can be either deterministic (typically partial differential equations, PDEs) or stochastic (often continuous-time Markov chains, CTMCs, or stochastic differential equations, SDEs) depending on whether the investigation focuses on population properties (deterministic methods) or individual paths (stochastic methods). Each state is commonly characterized in terms of a mean velocity, fluctuations about the mean velocity, and a distribution of time spent in the state, sometimes but not always determined by classical reaction rate theory. Explicit solutions for these models are rarely available, so asymptotic or numerical methods are often deployed to investigate and characterize the model’s predictions. The study of deterministic models often relies on numerical simulation using PDE integration methods (Wang et al. 2003; Cox and Matthews 2002; Trong et al. 2015), while stochastic models are simulated with Monte Carlo/Gillespie algorithms (Müller et al. 2008; Kunwar and Mogilner 2010; Müller et al. 2010; Allard et al. 2019) to generate individual trajectories that are then analyzed statistically. However, these computations can be quite costly, especially when one wants to understand how bulk transport properties (like effective velocity or diffusivity) depend on individual model parameters. When possible, asymptotic analysis allows for explicit approximation of transport properties, which can validate, complement, or even replace numerical simulations (Reed et al. 1990; Brooks 1999; Pavliotis 2005; Pavliotis and Stuart 2008; Popovic et al. 2011; McKinley et al. 2012; Bressloff and Xu 2015; Ciocanel et al. 2017).

The long-term effective velocity of state-switching particles is often straightforward to compute, usually obtained by calculating the fraction of time spent in each state and correspondingly averaging the associated state velocities. On the other hand, this weighted average technique is not valid when calculating a particle’s effective diffusivity, since this nonlinear quantity depends, via the Kubo formula (Kubo 1963), on the correlation of velocity at two different times. This in turn depends on the dynamics in the change in biophysical state beyond the stationary distribution of state. For example, randomness in the switching dynamics will produce a positive effective diffusivity even when the diffusivity within each state is zero. Generalizing some previous work (Brooks 1999; Hughes et al. 2011; Krishnan and Epureanu 2011; Hughes et al. 2012; Ciocanel et al. 2017), we consider this problem of computing effective diffusivity for a class of state-switching particle models that can be expressed in a framework where the sequence of states are given by a Markov chain, but the times spent in these states are not necessarily exponentially distributed as in a continuous-time Markov chain. Since we assume that the state process Markov chain is positive recurrent, the particle position process can be described as a regenerative increment process in a sense defined by Serfozo (2009), for example. That is to say, we consider processes that almost surely return to some base state at a sequence of (random) regeneration times such that the dynamics after a regeneration time are independent from those that occur before. As a result, we can decompose the process into what we refer to as *cycles*, in which the particle starts in a base state, undergoes one or more state changes, and then revisits the base state again. The dynamics within each cycle are independent of other cycles, and we can use the renewal reward theorem to perform asymptotic calculations by viewing the total displacement within each cycle as its reward and viewing the cycle durations as times between regenerations. An early application of the idea of computing effective particle velocity and diffusivity by decomposition and analysis of the dynamics in terms of independent cycles was to understand the large enhancement of (non-switching) particle diffusion in a tilted periodic potential (Reimann et al. 2001, 2002).

Our primary motivating examples are related to intracellular transport. Some prominent recent investigations include the study of mRNA localization in oocyte development (Zimyanin et al. 2008; Trong et al. 2015; Ciocanel et al. 2017), cell polarization in the budding yeast (Bressloff and Xu 2015), neurofilament transport along axons (Jung and Brown 2009; Li et al. 2014), interactions of teams of molecular motor proteins (Klumpp and Lipowsky 2005; Müller et al. 2008; Kunwar and Mogilner 2010; Müller et al. 2010; Tjioe et al. 2019), and sliding of parallel microtubules by teams of cooperative identical motors (Allard et al. 2019). Microtubule-based transport of cargo is typically mediated by kinesin motors moving material to the cell periphery and by dynein motors carrying it to the nucleus. Understanding population-scale behaviors, such as protein localization, that arise from local motor interactions remains an open question. While multiple motor interactions are usually thought to be resolved through a tug-of-war framework (Müller et al. 2008), it has been observed that important predictions made by the tug-of-war framework are not consistent with in vivo experimental observations (Kunwar et al. 2011; Hancock 2014). The work presented in this paper can aid theoretical efforts to relate local motor cargo dynamics to predictions for large-scale transport.

### PDE Methods for Markovian Switching

For hybrid switching diffusion processes (Yin and Zhu 2010), in which particles independently switch with continuous-time Markovian dynamics between states that have different velocities and/or diffusivities, the law of a particle can be expressed in terms of its associated forward Kolmogorov equations with an advection–reaction–diffusion structure:

1 $$\begin{aligned} \frac{\partial \varvec{{u}}(y,t)}{\partial t} = A^\mathrm{T}\varvec{{u}} - V \partial _y \varvec{{u}} + D \varDelta \varvec{{u}}\,. \end{aligned}$$

Here, we will think of $\varvec{{u}}$ as an $(N+1)$-dimensional column vector (indexed from 0 to *N*) of the concentrations of particle populations in different dynamical states, which also obey the forward Kolmogorov equations with a different normalization. The dynamics are governed by matrices $A, V, D \in {\mathbb {R}}^{(N+1) \times (N+1)}$, where *V* and *D* are diagonal matrices, with real constant diagonal entries $v_0, v_1,\ldots ,v_N$ for *V* corresponding to the particle velocities in each state, and positive real constant diagonal entries $d_0, d_1,\ldots ,d_N$ for *D* corresponding to the diffusion coefficients in each state. The matrix *A* is the transition rate matrix of the associated finite state continuous-time recurrent Markov chain (CTMC), *J*(*t*), which tracks the state of the particle at a given time. That is to say, each off-diagonal entry $a_{ij}$ can be interpreted as the rate at which a particle in state *i* switches to state *j*. The diagonal entries of *A* are non-positive and correspond to the total rate out of a given state. The rows of *A* sum to zero. Assuming that the CTMC is irreducible, it follows that *A* admits a zero eigenvalue with algebraic and geometric multiplicity one, and the corresponding normalized zero-eigenvector $\varvec{{\pi }}$ is the stationary distribution of *J*(*t*).

Either quasi-steady-state reduction (Bressloff and Newby 2013) or homogenization theory (Pavliotis and Stuart 2008) can be used to reduce the complexity of the advection–reaction–diffusion system (1) to a scalar advection–diffusion equation of the form:

$$\begin{aligned} \frac{\partial c(y,t)}{\partial t} = v_{\text {eff}} \partial _y c(y,t)+ D_{\text {eff}} \varDelta c(y,t)\,. \end{aligned}$$

with constant effective velocity $v_{\text {eff}}$ and constant effective diffusivity $D_{\text {eff}}$ for the particle concentration without regard to state $c(y,t) = \sum _{i=0}^N u_i (y,t)$. Quasi-steady-state reduction assumes the stochastic switching dynamics occurs on a fast scale relative to the advection–diffusion dynamics, while homogenization theory applies at sufficiently large space and time scales relative to those characterizing the dynamical scales. These different asymptotic conditions give in general distinct results when the transport and switching rates have explicit dependence on space, but when, as in the present case, they are spatially independent, the formulas for the effective transport coefficients coincide. (This is because the time scale of advection/diffusion is linked purely to the spatial scale, so the large spatial-scale assumption of homogenization will perforce induce a time-scale separation between the switching and transport dynamics, as assumed in quasi-steady-state reduction.) The effective velocity is computed by averaging the velocity in each state, weighted by the stationary distribution of the particle states:

2 $$\begin{aligned} v_{\text {eff}} = \varvec{{v}} \cdot \varvec{{\pi }}\,, \end{aligned}$$

where $\varvec{{v}} = (v_0,v_1,\ldots ,v_N)^\mathrm{T}$. The effective diffusivity is given, from an equivalent long-time effective dynamical description for intracellular transport derived by Lyapunov–Schmidt reduction on the low wavenumber asymptotics of the Fourier transform of Eq. (1) in Ciocanel et al. (2017, (2018), by

3 $$\begin{aligned} D_{\text {eff}}&= \varvec{{d}} \cdot \varvec{{\pi }} - \varvec{{v}} \cdot (\overline{A^\mathrm{T}})^{-1} (\varvec{{v}}\circ \varvec{{\pi }} -v_{\mathrm {eff}}\varvec{{\pi }}) \,, \end{aligned}$$

where

4 $$\begin{aligned} \varvec{{v}} \circ \varvec{{\pi }} = (v(0)\pi (0),v(1)\pi (1),\ldots ,v(N)\pi (N))^\mathrm{T} \end{aligned}$$

denotes the Hadamard product (component-wise multiplication of vectors). Here $\varvec{{d}} = (d_0,d_1,\ldots ,d_N)^\mathrm{T}$, and $\overline{A^\mathrm{T}}$ is the restriction of $A^\mathrm{T}$ to its range ${{\,\mathrm{Ran}\,}}(A^\mathrm{T})$ (vectors orthogonal to $(1,1,\ldots ,1)^\mathrm{T}$). Note that the operation involving the inverse of $(\overline{A^\mathrm{T}})^{-1}$ is well defined since $\overline{A^\mathrm{T}}$ is a full-rank matrix mapping ${{\,\mathrm{Ran}\,}}(A^\mathrm{T})$ to ${{\,\mathrm{Ran}\,}}(A^\mathrm{T})$, and its inversion in Eq. (3) is applied to a vector in ${{\,\mathrm{Ran}\,}}(A^\mathrm{T})$. We remark that the homogenization formula is often written (Cioranescu and Donato 1999; Pavliotis and Stuart 2008) in an equivalent adjoint form to Eq. (3), with a centering of the leading vector $\varvec{{v}} \rightarrow \varvec{{v}} - v_{\mathrm {eff}} (1,1,\ldots ,1)^\mathrm{T}$ that renders the formula indifferent to the choice of how to invert $A^\mathrm{T}$. The term $\varvec{{d}} \cdot \varvec{{\pi }}$ above reflects the contributions to the asymptotic diffusivity from pure diffusion, while the second term captures the interactions between the advection and reaction terms.

Applications of quasi-steady-state reduction to biophysical systems with state switching and diffusion can be found in Newby and Bressloff (2010b, (2010c), Bressloff and Newby (2011), Bressloff and Newby (2013), Bressloff and Xu (2015). Homogenization of Brownian motor models was conducted in Pavliotis (2005), Kramer et al. (2010).

### Summary of Method Based on Regeneration Cycles

These foregoing methods (Pavliotis and Stuart 2008; Ciocanel et al. 2017; Bressloff and Newby 2013) rely on the fully Markovian structure of the dynamics, with the state-switching process in particular taking the form of a continuous-time Markov chain with exponentially distributed state durations. In this work, we consider a generalized framework in which we require only that the sequence of states visited form a discrete-time recurrent Markov chain, but do not require exponentially distributed state durations, so the state-switching process *J*(*t*) need not be a continuous-time Markov chain. Moreover, we allow for more general random spatial dynamics within a state that also need not be fully Markovian. Our framework and method of analysis rather require only a regenerative structure of the dynamics, with repeated returns to a base state, at which moments the future randomness is decoupled from the previous history. For simplicity in exposition, we will restrict attention to the spatially homogeneous setup where all switching statistics and transport coefficients within each state are independent of spatial location, and comment in the discussion in Sect. 6 on how our results might extend to the spatially inhomogeneous context.

We use renewal reward theory and a functional central limit theorem to derive effective drift and diffusion for these more general switching diffusion systems in terms of the analysis of a single regeneration cycle. The calculation framework also results in an expression for the expected run length of cargo undergoing switching diffusion. Our approach builds on previous applications of renewal reward processes modeling motor-stepping and chemical cycles of bead-motor assays (Krishnan and Epureanu 2011; Hughes et al. 2011, 2012; Miles et al. 2018; Shtylla and Keener 2015) and extends the technique to accommodate more complex models with dynamics depending on the amount of time spent in the current state, as described in Sect. 2. Given the renewal reward framework, the analysis of the model reduces to computing the correlated spatial displacement and time duration of each cycle, which we study in Sect. 3.

We illustrate the usefulness of the probabilistic renewal reward techniques with several case studies. In Sect. 4, we show that our method of deriving effective velocity and diffusivity agrees with predictions in Ciocanel et al. (2017) arising from a Lyapunov–Schmidt reduction approach equivalent to homogenization for partial differential equations describing mRNA concentrations as in (1). In Sect. 5, we show that our method also agrees with previous theoretical and numerical analyses of transport properties for cargo pulled by teams of molecular motors. In the case of tug-of-war dynamics, with cargo transported by teams of opposite-directed motors, our framework provides predictions on the dependence of effective diffusivity on the ratio of stall to detachment force of the pulling motors. We also apply this method to a model accounting for increased reattachment kinetics when motors are already attached to the cargo and show that teams of opposite-directed motors have lower effective velocities but larger run lengths than teams consisting of the dominant motor only. Finally, we show that our effective diffusivity calculation agrees with stochastic simulations of sliding microtubule behavior driven by teams of bidirectional motors for a large range of load sensitivity. Since the method reduces to calculating the moments of times and spatial displacements in each dynamic state, this framework may also be useful for analyzing complex, higher-dimensional models [such as Bergman et al. (2018)], where cellular components interact in complicated ways, but moments of the dynamical changes in each state could be estimated numerically. As the experimental data on motor interactions develop rapidly, the framework proposed may prove useful in analyzing novel models and in understanding the dependence of effective transport properties on model parameters.

## Mathematical Framework and Examples

**Fig. 1:**
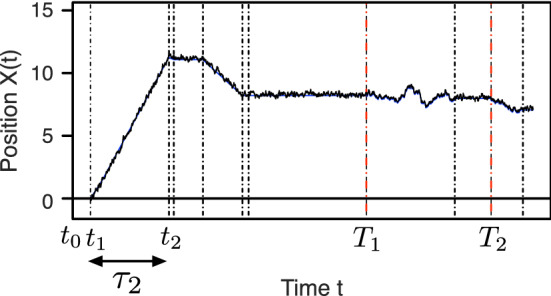
An example of the type of particle trajectory considered in this work. This simulated trajectory corresponds to the position of intracellular cargo (such as a vesicle) experiencing periods of active and diffusive transport. The path shown here illustrates switches between a forward transport state, a backward transport state, a stalled state, and a freely diffusing state in the framework of Eq. (5). The dashed vertical lines indicate random times $\{t_k : k \in {\mathbb {N}}\}$ when there are switches in the biophysical state. The base “renewal” state is free diffusion, and the red dashed vertical lines correspond to times $\{T_k : k \in {\mathbb {N}}\}$ when the system enters the base state. We denote the times spent in each state by $\tau _k$, as detailed in Sects. 2.1 and 2.2. In the language of the paper, the red lines correspond to the regeneration times and the total spatial displacements and times between these regeneration times are the “rewards” and the cycle durations, respectively (see Sect. 2.1)

The type of path we have in mind in this work is displayed in Fig. 1, a simulated continuous, stochastic process that switches between several stereotypical behaviors. Let the real-valued process $$\{X(t) : t \ge 0\}$$ be the time-dependent position of a particle and let $$\{J(t) : t \ge 0\}$$ denote the time-dependent underlying (e.g., biophysical) state, taking values from the finite state space $$S=\{0, 1, 2, \ldots , N\}$$. Switches between the states take place at the random times $$\{t_k : k \in {\mathbb {N}}\}$$, and we use $$\{J_k : k \in {\mathbb {N}}\}$$ to denote the state during the *k*th time interval $$[t_{k-1},t_k)$$. We set $$t_0=0$$ and $$J_1=J(0)$$. We assume that the sequence of states $$\{J_k : k \in {\mathbb {N}}\}$$ forms a time-homogeneous recurrent Markov chain with zero probability to remain in the same state on successive epochs. Given the state $$J_k$$, the associated state duration $$ t_{k}-t_{k-1}$$ and spatial displacement $$X(t_{k}) - X(t_{k-1})$$ are conditionally independent of all other random variables in the model (but not necessarily of each other). Moreover, the conditional joint distribution of $$ t_{k}-t_{k-1} $$ and $$ X(t_{k})-X(t_{k-1}) $$ given $$ J_k $$ depends only on the value of $$J_k$$ and not separately on the index *k*. In other words, the dynamics of (*J*(*t*), *X*(*t*)) have a statistically time-homogeneous character.

One general subclass of the processes considered can be expressed as follows: The random times $\{t_k\}_{k=0}^{\infty }$ are generated by sampling $t_{k}-t_{k-1}$ independently from their conditional marginal distributions given the Markov chain states $J_k$, and then conditioned upon these random variables, the spatial process *X*(*t*) is governed by a stochastic differential equation with coefficients depending on the current state, the value of *X* upon entry into the current state, and the time since entry into the current state. That is, we express the conditional dynamics of *X*(*t*) as:

5 $$\begin{aligned} \begin{aligned} \mathrm {d}X(t) = \sum _{k = 1}^\infty 1_{[t_{k-1},t_{k})} (t) \Big (\alpha _{J_k}\Big (X(t),X(t_{k-1}),t-t_{k-1}\Big ) \mathrm {d}t + \sqrt{2 d_{J_k}} \mathrm {d}W(t)\Big ), \end{aligned} \end{aligned}$$

where $\alpha _j : {\mathbb {R}}^2\times {\mathbb {R}}_+ \rightarrow {\mathbb {R}}$ is a function that describes the drift (deterministic component of the dynamics) of a particle while in the state *j*, and $d_j$ is the diffusivity in that state. (In general, the diffusive coefficients might also depend on the position of the particle and the recent history of the process, but we restrict ourselves to memoryless, additive noise for this discussion.)

Consider, for example, the stochastic process associated with the PDE (1), for which we set the drift terms in (5) to be $\alpha _j = v_j$, where $v_j$ is the constant *j*th diagonal entry of the velocity matrix *V* in (1). The diffusion coefficients would correspond to the entries of the diagonal matrix *D* in (1). The process would switch between states as a CTMC with rate matrix *A*, which, through standard theory (Lawler 1995, Sec. 3.2), induces a transition probability matrix for the sequence of states $\{ J_k : k \in {\mathbb {N}}\}$:

$$\begin{aligned} P_{ij}= {\left\{ \begin{array}{ll} \frac{A_{ij}}{{\bar{\lambda }}_i} &{} \text { for } i \ne j, \\ 0 &{} \text { for } i=j.\end{array}\right. } \end{aligned}$$

where ${\bar{\lambda }}_i \equiv \sum _{j \in S {\setminus } \{i\}} A_{ij}$ is the total transition rate out of state *i*. The state duration in state *i* would be exponentially distributed with mean ${\bar{\lambda }}_i^{-1}$.

We can articulate a more biophysically explicit model of motor cargo dynamics that takes into account, for example, the fluctuations of the cargo around the unobserved motor trajectory. Such a process is depicted in Fig. 1 and is inspired by data from kymograph readouts of cargo movement as seen in Encalada et al. (2011) and analyzed by a tool developed by Neumann et al. (2017). For the simulation depicted in Fig. 1, there are four states: a forward processive state, a backward processive state, and a stalled state—each of which is characterized by having a drift with speed $v_j$ plus Ornstein–Uhlenbeck type fluctuations [as described for example in Smith and McKinley (2018)]—and a freely diffusing state where the drift term equals zero. That is, $\alpha _0 = 0$ for the freely diffusing state and for $j > 0$,

6 $$\begin{aligned} \alpha _j(y,y_0,t) = -\frac{\kappa }{\gamma } \big (y - (v_j t + y_0)\big )\,, \end{aligned}$$

where $\kappa$ is a spring constant, $\gamma$ is the viscous drag, and $v_j$ is the velocity associated with the *j*th state. The term $(v_j t + y_0)$ indicates the theoretical position of a processive molecular motor that is simultaneously bound to the particle and to a microtubule.

### Remark 1

We note that there are at least two ways that the process *X*(*t*) can be considered to be non-Markovian and still fall within the set of models to which our results apply. The first, which is captured by the drift term (6), is that the process *X*(*t*) has memory in the sense that resolving *X*(*t*) on any interval in $$(t_k, t_{k+1})$$ depends on the value $$X(t_k)$$. A second allowable non-Markovian dynamic can be obtained by choosing the state duration times $$t_k - t_{k-1}$$ given state $$J_k $$ to have a non-exponential distribution. As long as the stochastic process of states $$\{J_k\}$$ is a time-homogeneous, positive recurrent Markov chain, the technique we present will apply.

In Sect. 2.3, we share a few examples from the molecular motors literature that include detailed assumptions about the set of achievable states and transitions among them. We note that these examples vary in their assumptions about fluctuations about mean behavior. In some cases, the dynamics are assumed to be “piecewise deterministic,” similar to the class of models studied by Brooks (1999) in which each state is characterized by a fixed velocity parameter $\alpha _j = v_j$ with the state diffusivity $d_j$ set to zero. In some of the other examples, fluctuations about the mean are included and would contribute to the long-term diffusivity as a result. Of course, fluctuations are always present in these dynamics (sometimes due to variability in the motor stepping, sometimes due to fluctuations in cargo position). There are natural ways to add these considerations to the models in Sect. 2.3 and express the dynamics within the framework of Eq. (5).

### Decomposition into Regenerative Cycles and Renewal Reward Structure

Here we outline our procedure for calculating the effective velocity and diffusivity of particles undergoing switching dynamics. The strategy is to break the process into independent “cycles” that are marked by returns to a chosen base state. As shown in Krishnan and Epureanu (2011), the analysis using the renewal reward structure is not affected by this initial choice of base state. An elementary exposition of this “dissection principle” concept can be found in Resnick (1992, Sec. 2.5). We define these times of re-entry into the base state as *regeneration times* $$\{T_n\}$$. In what follows, we will view the consecutive spatial displacements and time durations of the regenerative cycles to be the rewards and cycle durations of a classical renewal reward process (Cox 1962). Because the cycle statistics are independent and identically distributed after the first regeneration time $$ T_1$$, we define (in the sense of distribution) random variables for a generic cycle $$n\ge 2$$:

$$\begin{aligned} \begin{aligned} \varDelta X&{\mathop {=}\limits ^{{\mathcal {D}}}}X(T_{n}) - X(T_{n-1}); \qquad \varDelta T {\mathop {=}\limits ^{{\mathcal {D}}}}T_{n}-T_{n-1}; \quad \text { and} \\ M&{\mathop {=}\limits ^{{\mathcal {D}}}}\sup _{t\in [T_{n-1},T_{n}]} |X(t) - X(T_{n-1})|. \end{aligned} \end{aligned}$$

We rely on the functional central limit theorem (FCLT) presented in Serfozo (2009) for our asymptotic results. To this end, we define the quantities

$$\begin{aligned} \begin{aligned} \mu&:= {\mathbb {E}}(\varDelta T); \quad a := \frac{{\mathbb {E}}(\varDelta X)}{{\mathbb {E}}(\varDelta T)}; \text { and} \\ \sigma ^2&:= \text {Var}(\varDelta X - a \varDelta T). \end{aligned} \end{aligned}$$

As in previous work on molecular motor systems (Hughes et al. 2011, 2012), the FCLT justifies defining the effective (long-run) velocity and effective (long-run) diffusivity of the process *X*(*t*) in terms of properties of the regenerative increments as follows:

7 $$\begin{aligned} v_{\text {eff}}&:= \lim _{t \rightarrow \infty } \frac{1}{t} X(t) = a = \frac{{\mathbb {E}}(\varDelta X)}{{\mathbb {E}}(\varDelta T)}; \end{aligned}$$

8 $$\begin{aligned} D_\text {eff}&:= \lim _{t \rightarrow \infty } \frac{1}{2t} \text {Var}(X(t)) \nonumber \\&\,= \frac{\sigma ^2}{2 \mu } = \frac{1}{2 {\mathbb {E}}(\varDelta T)} \Big (\text {Var}(\varDelta X) + v_{\text {eff}}^2 \text {Var}(\varDelta T) - 2 v_{\text {eff}} \text {Cov}(\varDelta X, \varDelta T)\Big ). \end{aligned}$$

In more technically precise terms, the FCLT states: For $r \in {\mathbb {Z}}_+$, define $Y_r(t) := (X(rt) - art)/(\sigma \sqrt{r/\mu }).$ If $a, \mu , \sigma , {\mathbb {E}}(M)$, and $E((\varDelta T)^2)$ are all finite, then $\lim _{r \rightarrow \infty } Y_r = B$ in distribution for $t \in [0,1]$, where $\{B : t \in [0,1]\}$ is a standard Brownian motion (Whitt 2002).

### Notation for Events within Each Regeneration Cycle

The mathematical analysis in Sect. 3 focuses on calculation of the moments of the cycle duration and spatial displacement (reward) in an independent cycle of the process (introduced in Sect. 2.1). Here we introduce notation for events occurring within a single regeneration cycle. We denote the number of steps in the *n*th cycle by

$$\begin{aligned} \eta ^{(n)} := \min \{k \ge 1 : J_{k+K_{n-1}+1} = 0 \}, \end{aligned}$$

where $K_0 = 0$ and $K_n = \sum _{i = 1}^n \eta ^{(i)}$. We will let $\tau _{k}^{(n)} = t_{K_{n-1}+k}-t_{K_{n-1}+k-1}$ denote the times spent in each step of the *n*th cycle and $\xi _{k}^{(n)} = X(t_{K_{n-1}+k})-X(t_{K_{n-1}+k-1})$ denote the corresponding spatial displacements. The total time $\varDelta T$ and displacement $\varDelta X$ accrued in a cycle $n\ge 2$ before returning to the base state are then naturally the sum of these stepwise contributions:

9 $$\begin{aligned} \varDelta T := \sum _{k=1}^{\eta ^{(n)}} \tau _k^{(n)} \text { and } \varDelta X := \sum _{k=1}^{\eta ^{(n)}} \xi _k^{(n)}. \end{aligned}$$

In what follows, we drop the superscript denoting the index *n* of the cycle, since the cycles have statistically independent and identically distributed behavior for $n \ge 2$. We will decompose each cycle into what is accrued during the first step ($\tau _1$ and $\xi _1$) associated with the visit to the base state, and what accrues in all subsequent steps in the cycle, which we label

10 $$\begin{aligned} \varDelta {\tilde{T}} := \varDelta T - \tau _1 \text { and } \varDelta {{\tilde{X}}} := \varDelta X - \xi _1 \,. \end{aligned}$$

For each state $j \in S$ of the underlying Markov chain, let $\{\tau _k(j),\xi _k (j)\}_{k = 1}^\infty$ be a sequence of iid pairs of random variables drawn from the conditional joint distribution of durations and displacements occurring during a sojourn in state *j*. The rewards collected in each step can then be written as

$$\begin{aligned} \tau _k = \sum _{j=0}^{N}\tau _k(j) 1_{\{J_k = j\}} \text { and } \xi _k = \sum _{j=0}^{N}\xi _k(j) 1_{\{J_k = j\}} \,. \end{aligned}$$

In the statements of our main theorems, it will be useful to have a notation for a vector of random variables with distributions for the time durations and spatial displacements that are associated with the states $S = \{0, 1, \ldots , N\}$:

11 $$\begin{aligned} \varvec{{\tau }} {\mathop {=}\limits ^{{\mathcal {D}}}}(\tau (0),\tau (1),\ldots ,\tau (N)) \text { and } \varvec{{\xi }} {\mathop {=}\limits ^{{\mathcal {D}}}}(\xi (0),\xi (1),\ldots ,\xi (N)). \end{aligned}$$

So, for any step number $k \in {\mathbb {N}}$, we have that the vector $(\tau _k(0),\tau _k(1),\ldots ,\tau _k(N))$ is equal in distribution to the vector $\varvec{{\tau }}$ and likewise for the spatial displacements.

### Examples

The four-state example illustrated in Fig. 1 is just one of many models for intracellular transport that is carried out by multiple molecular motors. To provide context for this framework and for our result in Sect. 3, Proposition 1, here we introduce several canonical examples from the literature where intracellular transport of cargo can be modeled as a stochastic process with regenerative increments. Often, cargo fluctuations are neglected in models when a motor cargo complex is in a processing state (Müller et al. 2008, 2010; Kunwar and Mogilner 2010). This is equivalent to taking a limit in which the cargo is effectively instantaneously restored by the motor cargo tether to a fixed mechanical equilibrium configuration with respect to the motor.

#### Example 1

*(2-state advection–diffusion model of particle transport)*. Consider a 2-state advection–diffusion model for the dynamics of protein particles (such as mRNA) as illustrated in Ciocanel et al. (2017, Figure 3A), with a freely diffusing state and an active transport state. Assume that the times spent by the particles in each state are drawn from an exponential distribution

$$\begin{aligned} \tau (0)&\sim \text {Exp}(\beta _2)\,,\\ \tau (1)&\sim \text {Exp}(\beta _1)\,. \end{aligned}$$

Here $\beta _1$ and $\beta _2$ are the transition rates between states and the notation $\text {Exp} (r)$ denotes an exponential distribution with parameter *r* (equal to the inverse of the mean). The spatial displacement in each state is given by:

$$\begin{aligned} \xi (0)&= \sqrt{2D\tau (0)}Z \,,\\ \xi (1)&= v \tau (1)\,, \end{aligned}$$

where *D* is the diffusion coefficient in the freely diffusing state, *v* is the speed in the active transport state, and *Z* is an independent standard normal random variable.

#### Example 2

*(4-state advection–reaction–diffusion model of particle transport)*. More realistic representations of the dynamics of cellular protein concentrations lead to considering the more complex 4-state model illustrated in Ciocanel et al. (2017, Figure 3B), where particles may diffuse, move in opposite directions, or be paused. The state durations are exponentially distributed, with the switching rates between dynamical states provided in Ciocanel et al. (2017, Figure 3B), and the spatial displacements in each state are given by:

$$\begin{aligned} \xi (0)&= \sqrt{2D\tau (0)}Z \,,\\ \xi (1)&= v_+ \tau (1) \,,\\ \xi (2)&= v_- \tau (2) \,,\\ \xi (3)&= 0\,, \end{aligned}$$

with $v_+$ the particle speed in the forward active transport state and $v_-$ the particle speed in the backward active transport state.

#### Example 3

*(Cooperative models of cargo transport)*. Consider the cooperative transport models proposed in Klumpp and Lipowsky (2005); Kunwar and Mogilner (2010), where processive motors move cargo in one direction along a one-dimensional filament. These models assume a maximum number *N* of motor proteins, firmly bound to the cargo, that may act simultaneously in pulling the cargo in a specified direction (see Klumpp and Lipowsky (2005, Figure 1) for model visualization). The biophysical state (dynamic behavior) is defined by the number $0 \le n \le N$ of these motors that are bound to a filament and therefore actively contributing to transport. In a state with *n* motors attached to a filament, the cargo moves at a velocity $v_n$, motors can unbind from the filaments with rate $\epsilon _n$, or additional motors can bind to the filaments with rate $\pi _n$. The expressions for these transport model parameters are reproduced from Kunwar and Mogilner (2010), together with a nonlinear force–velocity relation:

12 $$\begin{aligned} v_n(F)&= v \left( 1-\left( \frac{F}{nF_s}\right) ^w \right) \,, \end{aligned}$$

13 $$\begin{aligned} \epsilon _n(F)&= n \epsilon e^{F/(nF_d)} \,, \end{aligned}$$

14 $$\begin{aligned} \pi _n&= (N-n)\pi \,. \end{aligned}$$

Here *v* is the load-free velocity of the motor, $\epsilon$ is the load-free unbinding rate, and $\pi$ is the motor binding rate. *F* is the externally applied load force, $F_s$ is the stall force, and $F_d$ is the force scale of detachment. The exponent *w* determines the nature of the force–velocity relation considered, with $w=1$ corresponding to a linear relation, $w<1$ corresponding to a concave sub-linear force–velocity curve, and $w>1$ corresponding to a convex super-linear force–velocity curve (Kunwar and Mogilner 2010). The times and displacements in each state *n* (with $0 \le n \le N$ motors bound to the filaments) are therefore given by:

15 $$\begin{aligned} \tau (n)&\sim \text {Exp}\big (r_{\mathrm {out}}(n)\big )\,, \nonumber \\ \xi (n)&= v_n(F) \tau (n) \,, \end{aligned}$$

where $r_{\mathrm {out}}(n) = \epsilon _n(F)+\pi _n$ is the transition rate out of the state with *n* motors [see Klumpp and Lipowsky (2005, Figure 1)].

#### Example 4

*(Tug-of-war models of cargo transport)*. Cargoes often move bidirectionally along filaments, driven by both plus and minus-directed motors. For example, kinesin moves cargo toward the plus end of microtubules while dynein moves it toward the minus end. In Müller et al. (2008, (2010), the authors propose a model where a tug-of-war between motors drives cargo in opposite directions, with transport by several motors leading to an increase in the time the cargo remains bound to a microtubule and is pulled along a particular direction. In these models, teams of maximum $$N_+$$ plus- and $$N_-$$ minus-end motors are bound to the cargo, and the biophysical state is given by the pair of indices $$ (n_+,n_-)$$ with $$ 0 \le n_+ \le N $$, $$ 0\le n_- \le N $$ indicating the number of plus and minus motors bound to the filament and thereby contributing actively to the transport (see Müller et al. (2008, Figure 1) for model visualization). A key assumption for this model is that motors interact when bound to the filament since opposing motors generate load forces, and motors moving in the same direction share the load. In addition, they assume that motors move with the same velocity as the cargo in any state (Müller et al. 2008, 2010). This model uses the following expressions for the transport parameters:

16 $$\begin{aligned} v_c(n_+,n_-)&= \frac{n_+F_{s+}-n_-F_{s-}}{n_+F_{s+}/v_{f+} + n_-F_{s-}/v_{b-}} \,, \end{aligned}$$

17 $$\begin{aligned} \epsilon _{+}(n_+)&= n_+ \epsilon _{0+} e^{F_c/(n_+F_{d+})} \,, \end{aligned}$$

18 $$\begin{aligned} \pi _+(n_+)&= (N_+-n_+)\pi _{0+} \,. \end{aligned}$$

Here indices $+$ and − refer to the plus- and minus-end directed motors under consideration. The model parameters are as follows: $F_s$ is the stall force, $F_d$ is the force scale for detachment, $\epsilon _0$ is the load-free unbinding rate, $\pi _0$ is the motor binding rate, $v_f$ is the forward velocity of the motor (in its preferred direction of motion), and $v_b$ is the slow backward velocity of the motor considered. Equation (16) applies for the case when $n_+F_{s+}>n_-F_{s-}$ [stronger plus motors, Müller et al. (2008)], and an equivalent expression with $v_{f+}$ replaced by $v_{b+}$ and $v_{b-}$ replaced by $v_{f-}$ holds for $n_+F_{s+}\le n_-F_{s-}$ (stronger minus motors). Equivalent expressions for the binding and unbinding rates hold for the minus-end directed motors. In the case of stronger plus motors, the cargo force $F_c$ when pulled by $n_+$ plus and $n_-$ minus motors is given by Müller et al. (2008):

$$\begin{aligned} F_c(n_+,n_-)&= \lambda n_+ F_{s+} + (1-\lambda )n_- F_{s-} \,, \nonumber \\ \lambda&= \frac{1}{1+ \frac{n_+F_{s+}v_{b-}}{n_-F_{s-}v_{f+}}}\,, \end{aligned}$$

with equivalent expressions for stronger minus motors as described above and in Müller et al. (2008). The times and displacements accumulated at each time step and in each state are defined as in Eq. (15) in Example 3.

## Analysis within a Single Cycle

From standard renewal reward and functional central limit theorem results, which we detailed in Sect. 2, we have related the computation of effective velocity and diffusivity via Eqs. (7) and (8) to analyzing the first and second moments and correlation of the spatial displacement and time spent in each regeneration cycle. In this section, the main result is Proposition 1, which provides these statistics. We begin with Lemma 1, by recalling a standard recursion formula for the moments of the reward accumulated until hitting a designated absorbing state. We include the proof of this lemma for completeness and as an example of the moment generating function approach we use in Lemma 2. In Proposition 1, we address the calculation of total displacement and time duration during the regeneration cycles described in Sect. 2.

Let 0 be the base state that marks the beginning of a new renewal cycle. We denote the set of remaining states as $S \backslash \{0\}$, and define ${\tilde{P}}$ as the $N \times N$ substochastic matrix containing the probabilities of transition among these non-base states only. Generally, we use the symbol ~  to refer to a vector or a matrix whose components corresponding to the base state have been removed.

Let *R* denote the total reward accumulated until the state process hits the base state. Note that the value of *R* will depend on what the initial state of the process is. In our motor transport examples, *R* corresponds to the time $\varDelta {\tilde{T}}$ or the displacement $\varDelta {\tilde{X}}$ accumulated after stepping away from the base state and before returning to the base state. Let $\rho _k$ denote the reward accumulated at each time step, recalling that time increments are denoted $\tau _k$ and displacement increments $\xi _k$ in Sect. 2.2.

By introducing random variables $\rho _k (j)$ for $j \in S$ and $k \in {\mathbb {N}}$ that indicate the reward received at step *k* if the particle is also in state *j* at that step, we can use indicator variables for the state to express: $\rho _k =\sum _{j=1}^N \rho _k(j) 1_{\{J_k = j\}}$ and

19 $$\begin{aligned} R = \sum _{k=1}^{\eta } \rho _k = \sum _{k=1}^{\eta } \sum _{j=1}^N \rho _k(j) 1_{\{J_k = j\}} \,. \end{aligned}$$

In the same way that we defined the distribution for the time durations and spatial displacements through the random vectors $\varvec{{\tau }}$ and $\varvec{{\xi }}$ in Eq. (11), we define the distribution of generic rewards through the vector of random rewards associated with each state:

20 $$\begin{aligned} \varvec{{\tilde{\rho }}} = (\rho (1), \rho (2), \ldots ,\rho (N)). \end{aligned}$$

The tilde notation is used here to be consistent with the connotation that tilde implies the zero state is excluded. When we need component-wise multiplication, we use the Hadamard power notation [see Eq. (4)]:

21 $$\begin{aligned} \varvec{{\tilde{\rho }}}^{\circ n} = (\rho ^n(1), \rho ^n(2),\ldots ,\rho ^n(N)). \end{aligned}$$

We define the moment-generating functions of the reward collected until the state process hits the base state, and of the reward in state *i*, respectively, by the following vectors:

22 $$\begin{aligned} \varvec{{\phi }}(s): \,\, \phi _i(s)&:= {\mathbb {E}}(e^{sR} \, | \,J_1=i)\,, \text {and} \nonumber \\ \varvec{{\psi }}(s): \,\, \psi _i(s)&:= {\mathbb {E}}(e^{s \rho (i)})\,. \end{aligned}$$

Characteristic functions could alternatively be used to handle rewards whose higher moments are not all finite; the results for the low order moments we calculate would be the same. Note that here and in the following, we will typically use index *i* to refer to states $i \in S \backslash \{0\}$. In Lemma 1, $J_1$ is the state in the initial step of the process. We seek a general recursion relation for ${\mathbb {E}}(R^n|J_1 = i)$ and denote the corresponding vector of moments for all $i \in S \backslash \{0\}$ by ${\mathbb {E}}_{S \backslash \{0\}}(R^n)$. The following result is a variation on similar recursion formulas for rewards accumulated in Markov chains (Hunter 2008; Palacios 2009).

### Lemma 1

Let $\{J_k\}_{k \ge 1}$ be a time-homogeneous, positive recurrent Markov chain with a transition probability matrix *P* (over a finite state space *S*) that has zeroes for each of its diagonal entries. Let the reward variables *R* and $\varvec{{{\tilde{\rho }}}}$ be defined as in Eqs. (19) and (20), respectively. For $n \in {\mathbb {N}}$, define the column vector

$$\begin{aligned} {\mathbb {E}}_{S {\setminus } \{0\}}(R^n) := \big (E(R^n \, | \,J_1 = 1), \, \ldots \, , E(R^n \, | \,J_1 = N)\big ). \end{aligned}$$

Then, this vector—the expected reward accumulated up to the first time that the state process $\{J_k\}$ hits the base state 0—satisfies the recursion relation

23 $$\begin{aligned} {\mathbb {E}}_{S \backslash \{0\}}(R)= & {} (I-{\tilde{P}})^{-1} {\mathbb {E}}(\varvec{{{\tilde{\rho }}}}); \nonumber \\ {\mathbb {E}}_{S \backslash \{0\}}(R^n)= & {} (I-{\tilde{P}})^{-1} \left( {\mathbb {E}}(\varvec{{{\tilde{\rho }}}}^{\circ n}) + \sum _{m=1}^{n-1} \left( {\begin{array}{c}n\\ m\end{array}}\right) \mathrm {diag}({\mathbb {E}}(\varvec{{{\tilde{\rho }}}}^{\circ (n-m)})) \, {\tilde{P}} \, {\mathbb {E}}_{S \backslash \{0\}}(R^m) \right) .\nonumber \\ \end{aligned}$$

Here ${\tilde{P}}$ is the substochastic matrix component of *P* excluding the base state 0, and $\varvec{{\tilde{\rho }}}^{\circ n}$ is the Hadamard *n*-th power vector defined in Eq. (21).

### Proof

Let *R*, the reward accumulated until hitting the base state 0, be decomposed into the reward from the first and from subsequent steps as follows: $R = \rho _1 + \check{R}$. We calculate the moment-generating function of *R* conditioned on the initial state $J_1 = i$ as follows:

24 $$\begin{aligned} \phi _i(s) :=&\, {\mathbb {E}}(e^{sR}\, | \,J_1=i) \nonumber \\ =&\, \sum _{j \in S} {\mathbb {E}}(e^{sR} \, | \,J_1=i,J_2=j) P_{ij} \nonumber \\ =&\, \sum _{j \in S} {\mathbb {E}}(e^{s\rho _1} e^{s\check{R}}\, | \,J_1=i,J_2=j) P_{ij}\nonumber \\ =&\, {\mathbb {E}}(e^{s \rho _1}\, | \,J_1=i) \left( {\mathbb {E}}(e^{s\check{R}}|J_2=0)P_{i0} + \sum _{j \in S \backslash \{0\}} {\mathbb {E}}(e^{s\check{R}} \, | \,J_2=j) P_{ij} \right) \nonumber \\ =&\, {\mathbb {E}}(e^{s \rho (i)}) \left( P_{i0} + \sum _{j \in S \backslash \{0\}} {\mathbb {E}}(e^{sR} \, | \,J_1=j) P_{ij} \right) \nonumber \\ =&\, \psi _i(s) \left( P_{i0} + \sum _{j \in S \backslash \{0\}} \phi _j(s) P_{ij} \right) , \end{aligned}$$

where $\psi _i(s)$ is defined in Eq. (22). In the fourth line, we used the Markov property, and in the fifth line, we used the fact that

$$\begin{aligned} (\check{R} \, | \,J_2=j) \sim (R \, | \,J_1=j)(1-\delta _{j0}) \end{aligned}$$

where $\delta _{ij}$ is the Kronecker delta function. Defining

$$\begin{aligned} f_i(s)&= \psi _i(s) P_{i0}\,, \; i \in S \backslash \{0\} \,,\\ G(s)&= \{ G(s,i,j); i,j \in S \backslash \{0\}: G(s,i,j)=\psi _i(s) P_{ij}\} \,, \end{aligned}$$

then we can write Eq. (24) in matrix vector form:

25 $$\begin{aligned} \varvec{{\phi }}(s)&= \varvec{{f}}(s) + G(s) \varvec{{\phi }}(s)\,. \end{aligned}$$

Since the moments of the reward before hitting the base state can be calculated using

$$\begin{aligned} {\mathbb {E}}(R^n\, | \,J_1=i) = \frac{\partial ^n}{\partial s^n} \phi _i(s)|_{s=0} \,, \end{aligned}$$

we calculate the derivatives:

$$\begin{aligned} \frac{\partial ^n \varvec{{\phi }}(s)}{\partial s^n}&= \frac{\partial ^n \varvec{{f}}(s) }{\partial s^n} + \sum _{m=0}^{n}\left( {\begin{array}{c}n\\ m\end{array}}\right) \frac{\partial ^{n-m} G(s) }{\partial s^{n-m}} \frac{\partial ^m \varvec{{\phi }}(s) }{\partial s^m} \,. \end{aligned}$$

For the first moment ($n=1$), each component yields:

$$\begin{aligned} \frac{\partial \phi _i(s)}{\partial s}&= P_{i0}{\mathbb {E}}(\rho _1\, | \,J_1=i) + \sum _{j \in S \backslash \{0\}} P_{ij} {\mathbb {E}}(\rho _1\, | \,J_1=i) + \sum _{j \in S \backslash \{0\}} P_{ij}\frac{\partial \phi _j(s)}{\partial s} \\&= {\mathbb {E}}(\rho (i)) + \sum _{j \in S \backslash \{0\}} P_{ij}\frac{\partial \phi _j(s)}{\partial s} \,. \end{aligned}$$

Evaluating at $s=0$ for $n=1$, we have

$$\begin{aligned} E(R \, | \,J_1 = i) = E(\rho (i)) + \sum _{j \in S {\setminus }\{0\}} P_{ij} E(R \, | \,J_n = j). \end{aligned}$$

Writing in vector form and solving for $E_{S {\setminus } \{0\}}(R)$ yield the first part of Eq. (23).

For higher-order moments ($n>1$):

$$\begin{aligned} \frac{\partial ^n \phi _i(s)}{\partial s^n}&= P_{i0}{\mathbb {E}}(\rho _1^n\, | \,J_1=i) + \sum _{j \in S \backslash \{0\}} P_{ij} {\mathbb {E}}(\rho _1^n\, | \,J_1=i) \\&\qquad + \sum _{m=1}^{n-1}\left( {\begin{array}{c}n\\ m\end{array}}\right) {\mathbb {E}}(\rho _1^{n-m}\, | \,J_1=i) \sum _{j \in S \backslash \{0\}} P_{ij} \frac{\partial ^m \phi _j(s)}{\partial s^m} + \sum _{j \in S \backslash \{0\}} P_{ij}\frac{\partial ^n \phi _j(s)}{\partial s^n} \\&= {\mathbb {E}}(\rho (i)^n) + \sum _{j \in S \backslash \{0\}} P_{ij}\frac{\partial ^n \phi _j(s)}{\partial s^n} \\&\qquad + \sum _{m=1}^{n-1}\left( {\begin{array}{c}n\\ m\end{array}}\right) {\mathbb {E}}(\rho (i)^{n-m}) \sum _{j \in S \backslash \{0\}} \!\! P_{ij} \frac{\partial ^m \phi _j(s)}{\partial s^m} \,. \end{aligned}$$

Evaluating at $s=0$ gives the recursion relation expressed in the second part of Eq. (23). $\square$

### Corollary 1

Let $\varvec{{\tau }}$ and $\varvec{{\xi }}$ denote the vectors of state-dependent time duration and spatial displacements as defined in Eq. (11). Let $\varDelta T$ and $\varDelta X$ denote the total time elapsed and displacement accumulated by a state-switching particle up until its state process $\{J_k\}_{k \ge 1}$ returns to the base state 0 [see Eqs. (9)]. Moreover, recall the first-step decomposition $\varDelta T = \tau _1 + \varDelta {{\tilde{T}}}$ and $\varDelta X = \xi _1 + \varDelta {{\tilde{X}}}$ (see Eqs. (10)). Suppose that the state process $\{J_k\}_{k \ge 1}$ and its associated transition probability matrix *P* satisfy the assumptions of Lemma 1. Then,

26 $$\begin{aligned} {\mathbb {E}}_{S \backslash \{0\}}(\varDelta {\tilde{T}})&= (I-{\tilde{P}})^{-1} {\mathbb {E}}(\varvec{{{\tilde{\tau }}}}) \,, \end{aligned}$$

27 $$\begin{aligned} {\mathbb {E}}_{S \backslash \{0\}}(\varDelta {\tilde{X}})&= (I-{\tilde{P}})^{-1} {\mathbb {E}}(\varvec{{{\tilde{\xi }}}}) \,, \end{aligned}$$

28 $$\begin{aligned} {\mathbb {E}}_{S \backslash \{0\}}(\varDelta {\tilde{T}}^2)&= (I-{\tilde{P}})^{-1} \left( {\mathbb {E}}(\varvec{{{\tilde{\tau }}}}^{\circ 2}) + 2 \mathrm {diag}({\mathbb {E}}(\varvec{{{\tilde{\tau }}}})){\tilde{P}} \, {\mathbb {E}}_{S \backslash \{0\}}(\varDelta {\tilde{T}}) \right) \, \end{aligned}$$

29 $$\begin{aligned} {\mathbb {E}}_{S \backslash \{0\}}(\varDelta {\tilde{X}}^2)&= (I-{\tilde{P}})^{-1} \left( {\mathbb {E}}(\varvec{{{\tilde{\xi }}}}^{\circ 2}) + 2 \mathrm {diag}({\mathbb {E}}(\varvec{{{\tilde{\xi }}}})){\tilde{P}} {\mathbb {E}}_{S \backslash \{0\}}(\varDelta {\tilde{X}}) \right) \,, \end{aligned}$$

where $\varvec{{{\tilde{\tau }}}}$ and $\varvec{{{\tilde{\xi }}}}$ are the vectors of time durations and spatial displacements excluding the base state.

### Proof

These results follow directly from Lemma 1, with $\varDelta {{\tilde{T}}}$ and $\varDelta {{\tilde{X}}}$, respectively, playing the role of the reward *R*. $\square$

### Lemma 2

Let $\varvec{{\tau }}$, $\varvec{{\xi }}$, $\varDelta {\tilde{T}}$, $\varDelta {\tilde{X}}$, *P*, and $\{J_k\}_{k \ge 1}$ be defined as in Corollary 1. Then,

30 $$\begin{aligned} {\mathbb {E}}_{S \backslash \{0\}}(\varDelta {\tilde{T}} \varDelta {\tilde{X}})&= (I-{\tilde{P}})^{-1}\left( {\mathbb {E}}(\varvec{{{\tilde{\tau }}}} \circ \varvec{{{\tilde{\xi }}}}) + \mathrm {diag}({\mathbb {E}}(\varvec{{{\tilde{\xi }}}})){\tilde{P}} {\mathbb {E}}_{S \backslash \{0\}}(\varDelta {\tilde{T}})\right. \nonumber \\&\qquad + \left. \mathrm {diag}({\mathbb {E}}(\varvec{{{\tilde{\tau }}}})){\tilde{P}} {\mathbb {E}}_{S \backslash \{0\}}(\varDelta {\tilde{X}}) \right) \,. \end{aligned}$$

### Proof

We use an argument similar to the re-arrangement of the moment-generating function in Eq. (25) in the proof of Lemma 1. Here we decompose the time and displacement into the first step after the base state and the subsequent steps: $\varDelta {\tilde{T}} = \tau _2 + \check{T}$ and $\varDelta {\tilde{X}} = \xi _2 + \check{X}$. Since we are interested in the cross-moment of the duration and displacement, we consider the following moment-generating function:

31 $$\begin{aligned} \phi _{i}(r,s)&= {\mathbb {E}}(e^{s \varDelta {\tilde{X}}}e^{r \varDelta {\tilde{T}}}|J_1 = 0, J_2 = i) \nonumber \\&= \sum _{j \in S} {\mathbb {E}}(e^{s \varDelta {\tilde{X}}} e^{r \varDelta {\tilde{T}}}|J_1=0,J_2 = i, J_3 = j) P_{ij} \nonumber \\&= \sum _{j \in S} {\mathbb {E}}(e^{s\xi _2}e^{r\tau _2} e^{s \check{X}}e^{r \check{T}}|J_2=i,J_3=j) P_{ij} \nonumber \\&= {\mathbb {E}}(e^{s\xi _2}e^{r\tau _2}|J_2=i) \sum _{j \in S} {\mathbb {E}}(e^{s \check{X}} e^{r \check{T}}|J_2=i,J_3=j) P_{ij} \nonumber \\&= {\mathbb {E}}(e^{s\xi (i)}e^{r\tau (i)}) \sum _{j \in S} {\mathbb {E}}(e^{s \check{X}} e^{r \check{T}}|J_3=j) P_{ij} \nonumber \\&= \psi _i(r,s) P_{i0} + \psi _i(r,s) \sum _{j \in S \backslash \{0\}} \phi _j(r,s) P_{ij} \,, \end{aligned}$$

where $$\psi _i(r,s) = {\mathbb {E}}(e^{s\xi (i)}e^{r\tau (i)})$$.

For the calculation of the cross-term ${\mathbb {E}}_{S \backslash \{0\}}(\varDelta {\tilde{T}} \varDelta {\tilde{X}})$, we note that $\frac{\partial ^2 \phi _i}{\partial r \partial s}|_{s=r=0} = {\mathbb {E}}(\varDelta {\tilde{T}} \varDelta {\tilde{X}}|J_2=i)$ and calculate:

$$\begin{aligned} \frac{\partial ^2 \phi _i}{\partial r \partial s}&= \frac{\partial ^2 }{\partial r \partial s} \left( \psi _i(r,s) \sum _{j \in S \backslash \{0\}} \phi _j(r,s) P_{ij} + \psi _i(r,s) P_{i0} \right) \nonumber \\&= \frac{\partial }{\partial r} \left( \frac{\partial \psi _i(r,s)}{\partial s} \sum _{j \in S \backslash \{0\}} \phi _j(r,s) P_{ij} + \psi _i(r,s) \sum _{j \in S \backslash \{0\}} \frac{\partial \phi _j(r,s)}{\partial s} P_{ij} + \frac{\partial \psi _i(r,s) }{\partial s} P_{i0} \right) \nonumber \\&= \frac{\partial ^2 \psi _i(r,s)}{\partial s \partial r} \sum _{j \in S \backslash \{0\}} \phi _j(r,s) P_{ij} + \frac{\partial \psi _i(r,s)}{\partial s} \sum _{j \in S \backslash \{0\}} \frac{ \partial \phi _j(r,s)}{\partial r} P_{ij} \nonumber \\&\qquad + \frac{\partial \psi _i(r,s)}{\partial r} \sum _{j \in S \backslash \{0\}} \frac{\partial \phi _j(r,s)}{\partial s} P_{ij} + \psi _i(r,s) \sum _{j \in S \backslash \{0\}} \frac{\partial ^2 \phi _j(r,s)}{\partial s \partial r} P_{ij} + \frac{\partial ^2 \psi _i(r,s) }{\partial s \partial r} P_{i0} \,. \end{aligned}$$

Evaluating the above at $s=r=0$ yields:

$$\begin{aligned} {\mathbb {E}}(\varDelta {\tilde{T}} \varDelta {\tilde{X}}|J_2=i)&= {\mathbb {E}}(\tau _2 \xi _2|J_2 = i) + {\mathbb {E}}(\xi _2|J_2 = i) \sum _{j \in S \backslash \{0\}} P_{ij} E(\varDelta {\tilde{T}}|J_2=j) \nonumber \\&\qquad \qquad + {\mathbb {E}}(\tau _2|J_2 = i) \sum _{j \in S \backslash \{0\}} P_{ij} {\mathbb {E}}(\varDelta {\tilde{X}}|J_2=j) \nonumber \\&\qquad \qquad + \sum _{j \in S \backslash \{0\}} P_{ij} {\mathbb {E}}(\varDelta {\tilde{T}} \varDelta {\tilde{X}}|J_2=j) \,. \end{aligned}$$

Therefore,

$$\begin{aligned} {\mathbb {E}}_{S \backslash \{0\}}(\varDelta {\tilde{T}} \varDelta {\tilde{X}})&= {\mathbb {E}}(\varvec{{{\tilde{\tau }}}} \circ \varvec{{{\tilde{\xi }}}}) + \mathrm {diag}({\mathbb {E}}(\varvec{{{\tilde{\xi }}}})){\tilde{P}} {\mathbb {E}}_{S \backslash \{0\}}(\varDelta {\tilde{T}}) \nonumber \\&\qquad + \mathrm {diag}({\mathbb {E}}(\varvec{{{\tilde{\tau }}}})){\tilde{P}} {\mathbb {E}}_{S \backslash \{0\}}(\varDelta X) + {\tilde{P}}{\mathbb {E}}_{S \backslash \{0\}}(\varDelta T \varDelta X), \end{aligned}$$

which yields Eq. (30). $\square$

### Remark 2

An alternative derivation of equation (30) would be to use a polarization argument for the expectation of the product:

$$\begin{aligned} {\mathbb {E}}_{S \backslash \{0\}}(\varDelta {\tilde{T}} \varDelta {\tilde{X}}) = \frac{1}{4} \left( {\mathbb {E}}_{S \backslash \{0\}}((\varDelta {{\tilde{X}}} + \varDelta {{\tilde{T}}})^2) - {\mathbb {E}}_{S \backslash \{0\}}((\varDelta {{\tilde{X}}}- \varDelta {{\tilde{T}}})^2)\right) \,. \end{aligned}$$

In this approach, the moment-generating function depending on both cycle time $\varDelta {{\tilde{T}}}$ and cycle displacement $\varDelta \tilde{X}$ introduced in Eq. (31) is not required, since Lemma 1 can be directly applied to give explicit formulas for the second moments of the reward $R=\varDelta {\tilde{X}} + \varDelta {\tilde{T}}$ and $R=\varDelta {\tilde{X}} - \varDelta {\tilde{T}}$.

We proceed to Proposition 1, which provides the quantities necessary to compute the effective velocity and diffusivity of the cargo dynamics using classical theory (see Eqs. (7) and (8) and the procedure in Sect. 2.1).

### Proposition 1

(First- and second-order statistics of rewards in a renewal cycle)

Consider a regenerative cycle of a discrete-time time-homogeneous recurrent Markov chain that takes its values in the discrete state space $S=\{0,1,2,\ldots ,N\}$ with probability transition matrix *P* with zero diagonal entries, starting at base state 0 until its first return to base state 0. The associated time $\varDelta T$ and spatial displacement $\varDelta X$ are defined as in Eq. (9). The random variables $\tau (0)$ and $\xi (0)$ have the distributions of the time duration and spatial displacement that are accumulated in the base state, and $\varvec{{p}}^{(1)}$ is the vector of transition probabilities from the base state in the first step of a cycle, i.e., the first row of *P*. The moments of the cycle time and displacement rewards are then given by:

32 $$\begin{aligned} {\mathbb {E}}(\varDelta T)&= {\mathbb {E}}(\tau (0)) + \varvec{{p}}^{(1)} \cdot {\mathbb {E}}_{S \backslash \{0\}}(\varDelta {\tilde{T}}) \,, \nonumber \\ {\mathbb {E}}(\varDelta X)&= {\mathbb {E}}(\xi (0)) + \varvec{{p}}^{(1)} \cdot {\mathbb {E}}_{S \backslash \{0\}}(\varDelta {\tilde{X}}) \,, \nonumber \\ \mathrm {Var}(\varDelta T)&= \mathrm {Var}(\tau (0)) + \varvec{{p}}^{(1)} \cdot {\mathbb {E}}_{S \backslash \{0\}}(\varDelta {\tilde{T}}^2) - (\varvec{{p}}^{(1)} \cdot {\mathbb {E}}_{S \backslash \{0\}}(\varDelta {\tilde{T}}))^2\,, \nonumber \\ \mathrm {Var}(\varDelta X)&= \mathrm {Var}(\xi (0)) + \varvec{{p}}^{(1)} \cdot {\mathbb {E}}_{S \backslash \{0\}}(\varDelta {\tilde{X}}^2) - (\varvec{{p}}^{(1)} \cdot {\mathbb {E}}_{S \backslash \{0\}}(\varDelta {\tilde{X}}))^2\,, \nonumber \\ \mathrm {Cov}(\varDelta X,\varDelta T)&= \mathrm {Cov}(\tau (0),\xi (0)) + \varvec{{p}}^{(1)} \cdot {\mathbb {E}}_{S \backslash \{0\}}(\varDelta {\tilde{T}} \varDelta {\tilde{X}}) \nonumber \\&\qquad - (\varvec{{p}}^{(1)} \cdot {\mathbb {E}}_{S \backslash \{0\}}(\varDelta {\tilde{T}}))(\varvec{{p}}^{(1)} \cdot {\mathbb {E}}_{S \backslash \{0\}}(\varDelta {\tilde{X}})) , \end{aligned}$$

where the first, second, and cross-moments of the time $\varDelta {{\tilde{T}}}$ and the displacement $\varDelta {{\tilde{X}}}$ are given by Eqs. (26), (27), (28), (29), (30) in Corollary 1 and Lemma 2.

### Proof

With state 0 as base state, we decompose the cycle time into the time spent in the base state $\tau _1 = \tau (0)$ and the time $\varDelta {\tilde{T}}$ spent from leaving the base state until returning to the base state. Therefore, the total time in a cycle is given by $\varDelta T = \tau _1 + \varDelta {\tilde{T}}$, and similarly, the total spatial displacement in a cycle is $\varDelta X = \xi _1 + \varDelta {\tilde{X}}$. We apply the law of total expectation by conditioning on the state $J_2$ that the process visits after the base state:

$$\begin{aligned} {\mathbb {E}}(\varDelta T)&= {\mathbb {E}}({\mathbb {E}}(\varDelta T|J_2))\nonumber \\&= \sum _{i \in S {\setminus } \{0\}} {\mathbb {E}}(\varDelta T|J_2 = i)P_{0i} \nonumber \\&= \sum _{i \in S {\setminus } \{0\}} {\mathbb {E}}(\tau _1 + \varDelta {{\tilde{T}}}|J_2 = i)P_{0i} \nonumber \\&= {\mathbb {E}}(\tau (0)) + \sum _{i \in S {\setminus } \{0\}} {\mathbb {E}}(\varDelta {{\tilde{T}}}|J_2 = i)P_{0i} \nonumber \\&= {\mathbb {E}}(\tau (0)) + \varvec{{p}}^{(1)} \cdot {\mathbb {E}}_{S {\setminus } \{0\}} (\varDelta {{\tilde{T}}}) \,, \end{aligned}$$

where as before $S \backslash \{0\}$ is the set of transient states and $P_{0i}$ is the probability of switching from base state 0 to state *i*. A similar calculation applies to the first moment of the cycle reward ${\mathbb {E}}(\varDelta X)$. For the second moments, we use the law of total variance as follows:

$$\begin{aligned} \mathrm {Var}(\varDelta T)&= {\mathbb {E}}(\mathrm {Var}(\varDelta T|J_2)) + \mathrm {Var}({\mathbb {E}}(\varDelta T|J_2)) \nonumber \\&= {\mathbb {E}}(\mathrm {Var}(\tau _1 + \varDelta {{\tilde{T}}}|J_2)) + \mathrm {Var}({\mathbb {E}}(\tau _1 + \varDelta {{\tilde{T}}}|J_2)) \nonumber \\&= {\mathbb {E}}(\mathrm {Var}(\tau (0)) + \mathrm {Var}( \varDelta {{\tilde{T}}}|J_2)) + \mathrm {Var}({\mathbb {E}}(\tau (0)) + {\mathbb {E}}(\varDelta {{\tilde{T}}}|J_2)) \nonumber \\&= \mathrm {Var}(\tau (0)) + {\mathbb {E}}(\mathrm {Var}( \varDelta {{\tilde{T}}}|J_2)) + \mathrm {Var}({\mathbb {E}}(\varDelta {{\tilde{T}}}|J_2)) \nonumber \\&= \mathrm {Var}(\tau (0)) + \sum _{i \in S{\setminus } \{0\}} \mathrm {Var}( \varDelta {{\tilde{T}}}|J_2=i) P_{0i} \nonumber \\&\qquad + \sum _{i \in S{\setminus } \{0\}} ({\mathbb {E}}(\varDelta {{\tilde{T}}}|J_2=i))^2 P_{0i} - \left( \sum _{i \in S{\setminus } \{0\}} {\mathbb {E}}(\varDelta {{\tilde{T}}}|J_2=i)P_{0i} \right) ^2 \nonumber \\&= \mathrm {Var}(\tau (0)) + \sum _{i \in S{\setminus } \{0\}} {\mathbb {E}}( \varDelta {{\tilde{T}}}^2|J_2=i) P_{0i} - \left( \sum _{i \in S{\setminus } \{0\}} {\mathbb {E}}(\varDelta {{\tilde{T}}}|J_2=i)P_{0i} \right) ^2 \nonumber \\&= \mathrm {Var}(\tau (0)) + \varvec{{p}}^{(1)} \cdot {\mathbb {E}}_{S {\setminus } \{0\}}(\varDelta {{\tilde{T}}}^2) -(\varvec{{p}}^{(1)} \cdot {\mathbb {E}}_{S {\setminus } \{0\}}(\varDelta {{\tilde{T}}}))^2 \,, \end{aligned}$$

and similarly for $\mathrm {Var}(\varDelta X)$. The covariance term can then be obtained via the polarization formula from the formulas for the variances.

$$\square $$

## Application to Models of Intracellular Transport

Proposition 1 and the calculation procedure in Sect. 2.1 can be applied to understand the long-term dynamics of protein intracellular transport described in Sect. 2 in Examples 1 and 2. The effective velocity and diffusivity of proteins are key in understanding large timescale processes such as mRNA localization in frog oocytes (Ciocanel et al. 2017) and cell polarization in the budding yeast (Bressloff and Xu 2015).

### 2-State Advection–diffusion Model of Particle Transport

In the following, we consider the 2-state transport-diffusion model for the dynamics of mRNA particles described in Example 1 and illustrated in Ciocanel et al. (2017, Figure 3A). We show how the calculations in Proposition 1 can be applied to determine the large-time effective velocity and diffusivity of the particles.

In the 2-state model, the probability transition matrix is simply $$P = \begin{pmatrix} 0 &{}\quad 1\\ 1 &{}\quad 0 \end{pmatrix}.$$ In this example, we take the diffusing state as the base state; however, our results are not dependent on this choice of base state as mentioned in Sect. 2.1. The substochastic matrix of the probabilities of transition between the other states (Bhat and Miller 2002) is then simply the scalar ${\tilde{P}} = 0$ in this case, while the vector of transitions out of the base state is simply $\varvec{{p}}^{(1)} = [1].$

The first and second moments of the cycle duration are given by Eqs. (26) and (28) with $(I-{\tilde{P}} )^{-1} = 1$. Similarly, the moments of the spatial displacement are given by Eqs. (27) and (29). In this model, we have that $S\backslash \{0\}=\{1\}$ and ${\tilde{\tau }}_k^{\circ n} = \tau _k^n(1)$ for the time reward and $\xi _k^{\circ n} = \xi _k^n(1)$ for the spatial displacement reward in the active transport state. In the 2-state system, these values are simply scalars:

$$\begin{aligned} \begin{aligned} {\mathbb {E}}_1(\varDelta {\tilde{T}})&= {\mathbb {E}}(\tau (1)) = 1/\beta _1 \,, \\ {\mathbb {E}}_1(\varDelta {\tilde{X}})&= {\mathbb {E}}(\xi (1)) = v/\beta _1 \,,\\ {\mathbb {E}}_1(\varDelta {\tilde{T}} \varDelta {\tilde{X}})&= {\mathbb {E}}(\tau (1) \xi (1)) = 2v/\beta _1^2 \,,\\ {\mathbb {E}}_1(\varDelta {\tilde{T}}^2)&= {\mathbb {E}}(\tau (1)^2) = 2/\beta _1^2\,,\\ {\mathbb {E}}_1(\varDelta {\tilde{X}}^2)&= {\mathbb {E}}(\xi (1)^2) = 2v^2/\beta _1^2 \,. \end{aligned} \end{aligned}$$

The statistics of the cycle are therefore given by:

$$\begin{aligned} \begin{aligned} {\mathbb {E}}(\varDelta T)&={\mathbb {E}}(\tau (0)) + {\mathbb {E}}_1(\varDelta {\tilde{T}}) \varvec{{p}}^{(1)}(1) = \frac{1}{\beta _2} + \frac{1}{\beta _1} \,, \\ {\mathbb {E}}(\varDelta X)&={\mathbb {E}}(\xi (0)) + {\mathbb {E}}_1(\varDelta {\tilde{X}}) \varvec{{p}}^{(1)}(1) = 0 + v/\beta _1 = \frac{v}{\beta _1} \,,\\ \text {Var}(\varDelta T)&= \text {Var}(\tau (0)) + {\mathbb {E}}_1(\varDelta {\tilde{T}}^2) \varvec{{p}}^{(1)}(1) - ({\mathbb {E}}_1(\varDelta {\tilde{T}}) \varvec{{p}}^{(1)}(1))^2 = \frac{1}{\beta _2^2} + \frac{1}{\beta _1^2}\,,\\ \text {Var}(\varDelta X)&= \text {Var}(\xi (0)) + {\mathbb {E}}_1(\varDelta {\tilde{X}}^2) \varvec{{p}}^{(1)}(1) - ({\mathbb {E}}_1(\varDelta {\tilde{X}}) \varvec{{p}}^{(1)}(1))^2 = \frac{2D}{\beta _2} + \frac{v^2}{\beta _1^2} \,,\\ \text {Cov}(\varDelta T,\varDelta X)&= \text {Cov}(\tau (0),\xi (0)) + {\mathbb {E}}_1(\varDelta {\tilde{T}} \varDelta {\tilde{X}}) \varvec{{p}}^{(1)}(1) \\&\qquad - \left( {\mathbb {E}}_1(\varDelta {\tilde{T}}) \varvec{{p}}^{(1)}(1)\right) \left( {\mathbb {E}}_1(\varDelta {\tilde{X}}) \varvec{{p}}^{(1)}(1)\right) = \frac{v}{\beta _1^2}\,. \end{aligned} \end{aligned}$$

Equations (7) and (8) then provide expressions for the effective velocity and diffusivity of the particles as in Hughes et al. (2011, (2012), Whitt (2002):

$$\begin{aligned} v_{\mathrm {eff}}&= \frac{{\mathbb {E}}(\varDelta X)}{{\mathbb {E}}(\varDelta T)} = v \frac{\beta _2}{\beta _1+\beta _2}\,, \nonumber \\ D_{\mathrm {eff}}&= \frac{1}{2 {\mathbb {E}}(\varDelta T)} (v_{\mathrm {eff}}^2 \text {Var}(\varDelta T) + \text {Var}(\varDelta X) - 2v_{\mathrm {eff}}\text {cov}(\varDelta T,\varDelta X)) \nonumber \\&= D\frac{\beta _1}{\beta _1+\beta _2} + v^2 \frac{\beta _1 \beta _2}{(\beta _1+\beta _2)^3} \,. \end{aligned}$$

Note that the effective velocity is given by the speed in the transport state multiplied by the fraction of time the mRNA particles spend in the moving state. The effective diffusivity has a more complicated expression, but clearly shows the dependence of this quantity on each model parameter. These expressions agree with the results of Eqs. (2), (3) as outlined in Ciocanel (2017).

### 4-State Advection–Reaction–Diffusion Model of Particle Transport

Our calculation procedure and Proposition 1 extend to more complicated and realistic models such as the 4-state model described in Example 2 and illustrated in Ciocanel et al. (2017, Figure 3B). By considering the stochastic transitions between dynamic states and the durations and displacements accumulated in each state, the effective velocity and diffusion of cargo can be calculated in an intuitive way even for such complex models with many transition states. Since this approach requires calculating the inverse of the invertible matrix $I-{\tilde{P}}$ (see Bhat and Miller 2002; Dobrow 2016) to determine the fundamental matrix, the approach presented here is easily implemented in a software package such as *Mathematica* or *MATLAB* for symbolic derivation of the effective transport properties for models with multiple states [see sample code in the repository on GitHub (2019)].

**Fig. 2:**
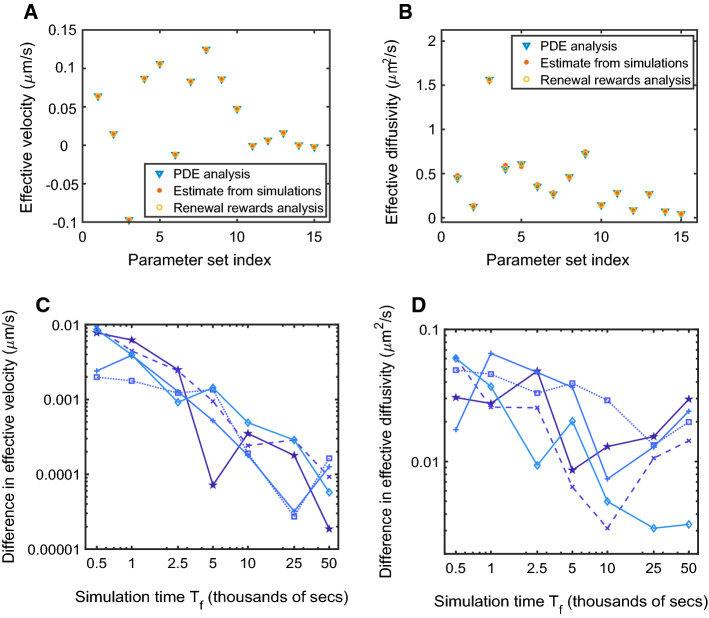
**a**, **b** Effective velocity (**a**) and effective diffusivity (**b**) of particles switching between diffusion, bidirectional transport, and stationary states as in (Ciocanel et al. 2017, Figure 3B) for different parameter sets. Blue triangles correspond to predictions based on the homogenization or equivalent analysis (Ciocanel et al. 2017) of the corresponding PDEs [Eq. (1)], filled red dots correspond to estimates from multiple simulated realizations of the Markov chain, and yellow circles correspond to predictions based on analysis of the corresponding renewal process model combined with Proposition 1. **c**, **d** Difference between effective velocity (**c**) and effective diffusivity (**d**) as computed by renewal reward asymptotics and Monte Carlo simulations over a finite time $T_f$. Results from five of the parameter sets from (**a**, **b**) are shown, and the axes are in log scale

In Fig. 2, we illustrate the good agreement of the results in Ciocanel et al. (2017) with our calculation procedure in Sect. 2.1 [Proposition 1 combined with Eqs. (7) and (8)] based on 15 sets of parameters estimated in Ciocanel et al. (2017). In addition, we validate results from both approaches by carrying out numerical simulations of the particle transport process and empirically estimating the effective transport properties. In particular, we set up a Markov chain of the 4-state model in Ciocanel et al. (2017, Figure 3B). For each parameter set, we consider $N_R = 500$ stochastic realizations of the dynamics, and for each iteration, we run the process until a fixed large time $T_f = 5\times 10^4$, which keeps the computation feasible. We then estimate the effective velocity and diffusivity as follows:

$$\begin{aligned} v_{\mathrm {eff}}&\approx \frac{(\sum _{i=1}^{N_R} X_i(T_f))/N_R}{T_f}\,, \\ D_{\mathrm {eff}}&\approx \frac{\left( \sum _{i=1}^{N_R}(X_i(T_f) - (\sum _{i=1}^{N_R}X_i(T_f))/N_R)^2\right) /(N_R-1)}{2T_f}\,, \end{aligned}$$

where $X_i(T_f)$ are the simulated final positions of the particle at time $T_f$ in iteration *i*.

The different parameter sets (labeled by index) in Fig. 2a–b correspond to simulations using parameter estimates based on FRAP mRNA data from different frog oocytes in Gagnon et al. (2013), Ciocanel et al. (2017). The good agreement of the theoretical and simulated effective velocity and diffusivity shows that the analytical approach proposed is a good alternative to potentially costly simulations of the stochastic process up to a large time.

The theoretical formulas for effective velocity and diffusivity are long-time asymptotic results, which raises the question of how well they apply at finite times. In Fig. 2c, d, we show the difference between the predicted and simulated effective velocity and diffusivity for each parameter set as a function of simulation time $T_f$. The convergence rate for the renewal reward asymptotic theory relies on a large number of regeneration cycles. We can estimate the number of regeneration cycles in a simulation as $T_f/{\mathbb {E}}(\varDelta T)$, with the expected cycle time ${\mathbb {E}}(\varDelta T )$ computed for each parameter set from Eq. (32). For the five parameter sets used in Fig. 2c, d, the cycle time ${\mathbb {E}}(\varDelta T )$ is given by: 114s, 477s, 135s, 175s, and 747s. Across the 15 parameter sets considered, we found that 10 regeneration cycles were usually sufficient for the simulated finite-time velocity and diffusivity to be within 10% of the theoretical asymptotic value.

## Application to Cooperative and Tug-of-War Models of Cargo Transport

The framework presented here also extends to models of cargo particles driven by changing numbers of motor proteins. The analytical calculation of transport properties of cargo pulled by motors in the same or opposite directions could replace or complement costly numerical simulations of individual cargo trajectories. In the following, we consider both models of cooperative cargo transport with identical motors (Klumpp and Lipowsky 2005; Kunwar and Mogilner 2010) and tug-of-war models of bidirectional transport driven by identical or different motors moving in opposite directions (Müller et al. 2008, 2010). While not discussed here, this framework may also prove useful in analyzing stochastic models of nanoparticulate transport in biogels, where states correspond to the number of occupied binding sites on nanoparticulates and to the number of molecular anchors crosslinking them to the matrix of polymers (Newby et al. 2017).

### Cooperative Models of Cargo Transport

We start by considering the cooperative transport models described in Sect. 2, Example 3, and studied by Klumpp and Lipowsky (2005); Kunwar and Mogilner (2010), with processive motors that move along a one-dimensional microtubule and transport cargo in only one direction. The cargo movement is described in terms of the force-driven velocities $v_n$, unbinding rates $\epsilon _n$, and binding rates $\pi _n$ in each state with *n* motors simultaneously bound to the cargo and the microtubule [see Eqs. (12), (13), and (14)]. In this section, we use the kinetic parameters for conventional kinesin-1 provided in Klumpp and Lipowsky (2005) (see Table 1 in “Appendix”).

Our calculation of the effective velocity of cargo agrees with the derivation in Klumpp and Lipowsky (2005), which uses the stationary solution of the master equation for probabilities of the cargo being in each state (i.e., carried by *n* motors). We note that there are two notions of effective velocity (and diffusivity) that can be used in studying this model: one is to calculate the effective velocity of the cargo averaged over the bound states only (the asymptotic velocity without detachment along a theoretical infinite length microtubule) (Klumpp and Lipowsky 2005; Kunwar and Mogilner 2010), and the second is to calculate the overall effective velocity that also accounts for periods of detachment from microtubules. For the $N=2$ motors model, Klumpp and Lipowsky (2005) and Kunwar and Mogilner (2010) report the average velocity for bound cargo (first notion):

33 $$\begin{aligned} v_{\mathrm {eff}} = v_1 \frac{\pi _0 \epsilon _2}{\pi _0 \epsilon _2+\pi _0\pi _1} + v_2 \frac{\pi _0 \pi _1}{\pi _0 \epsilon _2+\pi _0\pi _1}\,. \end{aligned}$$

Since we are interested in the overall effective velocity of the particles in the context of their full dynamics, we include the state where no motors are bound to the filament in our calculation, so that the effective velocity with respect to the overall dynamics is given by:

34 $$\begin{aligned} v_{\mathrm {eff}}&= v_1 \frac{\pi _0 \epsilon _2}{\epsilon _1\epsilon _2 + \pi _0 \epsilon _2+\pi _0\pi _1} + v_2 \frac{\pi _0 \pi _1}{\epsilon _1\epsilon _2 + \pi _0 \epsilon _2+\pi _0\pi _1}\,. \end{aligned}$$

**Fig. 3:**
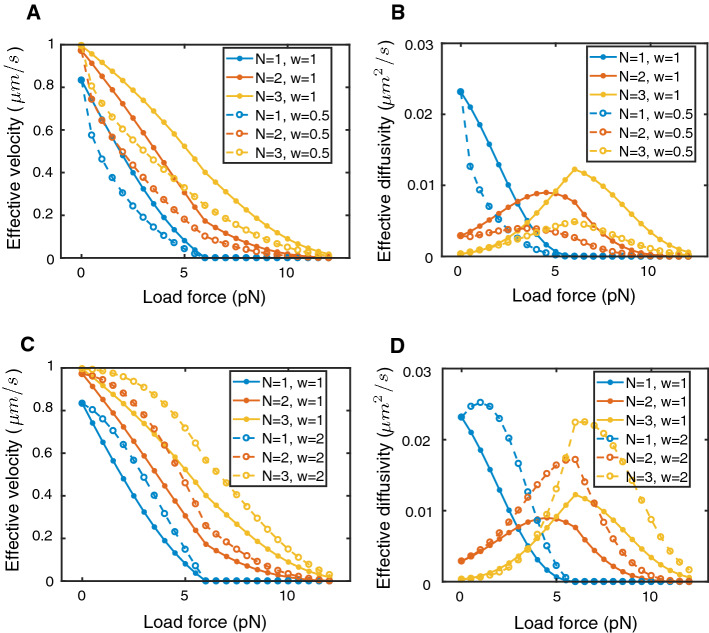
Effective velocity (**a**, **c**) and effective diffusivity (**b**, **d**) of cargo driven by a maximal number *N* of forward motor proteins as a function of the load force under various force–velocity exponents *w* (Eq. (12)). The stall force used for kinesin is $F_s = 6$ pN (see Table 1 in Appendix for all parameters). Solid lines correspond to motors with a linear force–velocity relation, and dashed lines correspond to sub-linear motors with a convex-up velocity–force relation (top row) and, respectively, to super-linear motors with a concave force–velocity relation (bottom row)

Using the calculation of the overall effective velocity in (34), we predict a similar dependence of the effective velocity under a range of force loads as in Klumpp and Lipowsky (2005) using the formula (33). The dashed curves in Fig. 3a, c agree with the behavior of sub- and super-linear motors under different load forces as reported in Kunwar and Mogilner (2010, Figure 2C-D), including the fact that sub-linear motors have lower effective velocities for any choice of the load force and for all maximum motor numbers *N* considered (A, $w=0.5$), while super-linear motors are faster and therefore have larger effective velocities than linear motors (C, $w=2$).

The insight from our method lies in the prediction of the effective diffusivity as a function of load for each type of motor. Figure 3b, d shows that the $N=1$ motor transport case has a large effective diffusivity under no load because of the switching between the paused and moving states. As the force load increases to stall $F_s$, the velocity of the single motor state decreases to 0: $v_1(F) = v\left( 1-F/F_s \right)$. Therefore, the active transport state switches to a stationary state at $F=F_s=6$ pN, leading to decreased effective diffusivity as the cargo switches between dynamic states with similar behaviors.

For $N=2$ and $N=3$, the calculation of the effective diffusivity allows us to re-visit the cooperative transport models for a large range of load forces and observe a new phenomenon in the classical models of Klumpp and Lipowsky (2005), Kunwar and Mogilner (2010). The broader sweep of the load force parameter in Fig. 3b, d shows a non-monotonic dependence of the effective diffusivity on load force for all types of motors considered (linear, sublinear, and superlinear), with an increase in effective diffusivity of cargo at low load forces and a decrease at large load forces. While it is not immediately clear what leads to this phenomenon, we conjecture that this observation may be a result of the balance between two competing effects: on the one hand, as the load increases, there is more detachment of motors [see (13)] and thus more frequent switches between transport and stationary states, leading to an increase in effective diffusivity; on the other hand, the increase in load force leads to a decrease in the speeds of the motor-driven cargo states [see (12)] and thus a decrease in effective diffusivity.

### Tug-of-War Models of Cargo Transport

In Example 4 in Sect. 2, we consider the case where plus- and minus-directed motors can drive cargo bidirectionally along filaments. The cargo velocities $v_c(n_+,n_-)$, unbinding rates $\epsilon _{+/-}(n_+,n_-)$, and binding rates $\pi _{+/-}(n_{+/-})$ depend on the number of plus motors $n_+$ and minus motors $n_-$ at each state.

**Fig. 4:**
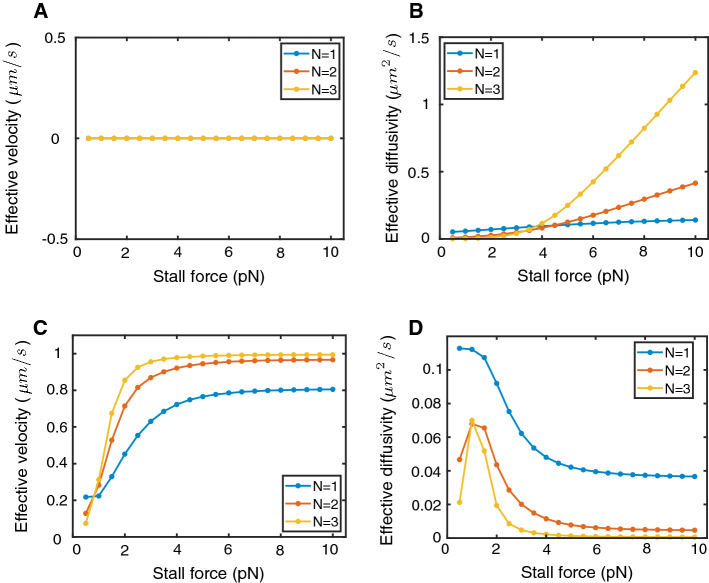
Effective velocity (**a**, **c**) and effective diffusivity (**b**, **d**) of cargo driven by maximum *N* forward and maximum *N* backward motor proteins as a function of kinesin-1 stall force $F_s$; the detachment force is $F_d = 3$ pN (see Table 1 in “Appendix” for all parameters). Panels (**a**, **b**) correspond to identical forward and backward motors with kinetic parameters for kinesin-1, and panels (**c**, **d**) correspond to kinesin-1 forward motors and conventional dynein backward motors

*Identical plus and minus motors.* With kinesin parameters drawn from Müller et al. (2008) (see Table 1 in “Appendix”), we first calculate the transport properties of cargo in these models for identical plus and minus motors in equal numbers ($N_+=N_-$). We vary the stall force of the kinesin motor to determine if the theoretical effective velocity and diffusivity capture the differences obtained in the numerical simulation studies in Müller et al. (2008, (2010) for weak motors (small stall to detachment force ratio $f = F_s/F_d$) and strong motors (large *f*). As expected, the effective velocity in this symmetric case of identical motors is zero for all stall forces (see Fig. 4a). The predicted effective diffusivity in Fig. 4b shows that for weak motors, the effective diffusivity is small, and different maximum numbers of motors do not lead to significant differences. This is similar to the results in Müller et al. (2008), where the simulated cargo trajectories show small fluctuations and the probability distribution for the velocity has a single maximum peak corresponding to approximately equal numbers of plus and minus motors attached. However, for strong motors with a larger stall to detachment force ratio, the effective diffusivity increases considerably for all models. This is consistent with the observation in Müller et al. (2008) that strong motors lead to cascades of unbinding of minus motors until only plus motors stay bound (and vice versa), so that the spread of the cargo position is predicted to be larger. The larger motor numbers lead to a more significant increase in effective diffusivity as observed in Müller et al. (2010), where the simulated diffusion coefficient grows exponentially with motor numbers and therefore leads to a more productive search of target destinations throughout the domain (Müller et al. 2010).

It is worth noting that the method we develop in Sect. 2.1 extends to cases where slow diffusive transport rather than pausing is observed in the unbound state (see Sect. 4 for another example with a diffusive state). As expected, when the cargo has an intrinsic diffusion coefficient that is nonzero, the effective velocity of the cargo does not change; however, the effective diffusivity is consistently larger than in the case where the unbound cargo is fully stationary (results not shown).

*Distinct plus and minus motors.* When considering dynein as the minus-end directed motor in the bidirectional transport model, we use the kinetic parameters estimated to fit *Drosophila* lipid droplet transport in Müller et al. (2008) (see Table 1 in “Appendix”). Figure 4c shows that the cargo is predicted to move in the forward (kinesin-driven) direction with a positive effective velocity. We again observe increased transport efficiency for larger numbers of motors. With increasing stall force, the velocity of individual runs in each state increases, and therefore, the effective velocity increases and then plateaus. This asymmetric motor case also results in effective diffusivity that decreases past a small stall force and then stabilizes (see Fig. 4d). Since the kinesin motor dominates the dynamics, there are fewer excursions backwards than in the case of identical motors, so that the effective diffusivity is an order of magnitude smaller. Larger teams of motors regularize the dynamics and display decreased effective diffusivity. We remark that an asymmetric tug-of-war may even occur in motor interactions of a single type, as recently observed in force-gliding assay experiments of kinesins moving microtubule cargoes in Tjioe et al. (2019). The analysis proposed here could be applied to models accounting for switching between different behaviors (states) of kinesin motors, such as “driving” kinesins pulling on a microtubule and “resisting” kinesins holding it back (Tjioe et al. 2019).

**Fig. 5:**
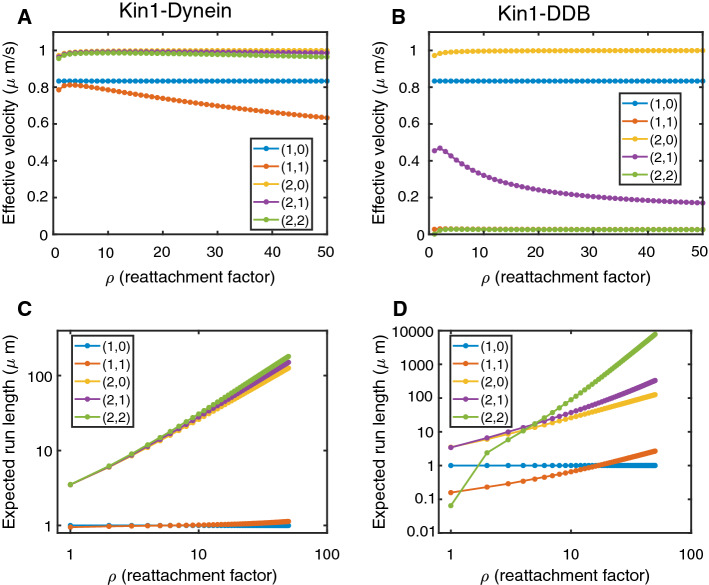
Effective velocity (**a**, **b**) and expected run length (**c**, **d**) of cargo driven by maximum $N_1$ forward (kinesin-1) and maximum $N_2$ backward (dynein) motor proteins $(N_1,N_2)$ as a function of the reattachment factor $\rho$. Panels (**a**, **c**) use conventional dynein motor kinetics as in Müller et al. (2008, (2010) while panels (**b**, **d**) use dynein–dynactin–BicD2 (DDB) complex parameters as in Ohashi et al. (2019); see Table 2 in “Appendix” for all parameters. The run lengths in panels **b** and **d** are plotted on a log–log scale to allow for visualization of differences between the models considered. The definitions of effective velocity and run length used are provided in the text

### Reattachment in Models of Cargo Transport

In vitro experiments have suggested that binding rates of molecular motors at specific locations may be regulated by the concentration of the same or opposite-directed motors (Hancock 2014), as well as by the availability of microtubule filaments. To test for the impact of reattachment kinetics in the standard transport models of Müller et al. (2008, (2010), we modify the binding rate in (18) to account for a higher likelihood of reattachment when a motor (of either type) is already attached to the microtubule:

$$\begin{aligned} \pi _+(n_+,n_-)&= {\left\{ \begin{array}{ll} N_+\pi _{0+}, \quad \mathrm {\, \, if \, \, n_++n_-=0} \,,\\ (N_+-n_+)\rho \pi _{0+}, \quad \mathrm {\, \, \, \, else} . \end{array}\right. } \end{aligned}$$

Here $\rho >0$ denotes the reattachment factor, and an equivalent expression is valid for the binding rate for minus motors $\pi _-(n_+,n_-)$. $\rho =1$ corresponds to the binding kinetics in the previous sections, and $\rho >1$ denotes an increased reattachment likelihood when other motors are attached.

Figure 5 illustrates the effective velocity (panels a,b) and the expected cargo run length (Eq. (32) for $\varDelta X$, panels c,d) for values of $\rho$ ranging from 1 to 50, in the context of models labeled $(N_1,N_2)$ with transport driven by maximum $N_1$ forward (kinesin-1) motors and $N_2$ backward (dynein) motors. Here we report the overall effective velocity of the cargo according to the second definition in Sect. 5.1, which includes both attached and detached cargo states in the calculation. In addition, the mean run length is calculated as the mean total displacement over a cycle starting with all motors detached until its return to a completely detached state, namely ${\mathbb {E}}(\varDelta X)$ in Eqs. (32). Note the base state of complete detachment makes no contribution to the mean displacement.

Classically in tug-of-war modeling, dynein has been viewed as a “weaker partner” than kinesin family motors. In this parameter regime (Müller et al. 2008, 2010), dynein has both a smaller stall force and smaller critical detachment force than kinesin-1. As a result, when equal numbers of kinesin-1 and dynein are simultaneously attached, kinesin-1 dominates transport. However, it has recently been shown that it might not be realistic to consider dynein in the absence of its helper proteins, particularly dynactin and BicD2. Together, these form a complex referred to as DDB, and the associated parameter values (Ohashi et al. 2019) are much more “competitive” with kinesin-1 in a tug-of-war scenario (see Table 2 in “Appendix”).

In Fig. 5, we display the effective velocity and expected run length of kinesin-1 vs dynein (panels a,c), and kinesin-1 versus DDB (panels b,d) dynamics. In Fig. 5a, the effective velocity of cargo driven by teams of motors approaches the effective speed predicted for kinesin-only motor teams (models (1, 0) and (2, 0)) for small values of $\rho$, but then decreases as $\rho$ becomes larger for conventional dynein motility. As observed in recent studies, activated dynein competes more efficiently with kinesin, and therefore, the teams of opposite-directed motors are consistently slower than teams consisting of only the forward kinesin motor protein in Fig. 5b (Ohashi et al. 2019). The expected run lengths in Fig. 5c, d illustrate that teams of multiple motors are characterized by significantly increased processivity on microtubules as the reattachment factor becomes larger. When considering conventional dynein, the difference in processive cargo motion between the cooperative and tug-of-war models is only observed at large values of the reattachment constant $\rho$ ($>10$, see Fig. 5c). This is due to the fact that the backward motor (conventional dynein) in the Müller et al. (2008) model is weak with a small detachment force, so that overcoming the large dynein unbinding rate requires large values of the reattachment factor. On the other hand, activated dynein in the DDB complex is a more equal competitor to kinesin, with predictions of the expected run length in Fig. 5d confirming the experimental observations of larger unloaded run lengths in Ohashi et al. (2019).

**Fig. 6:**
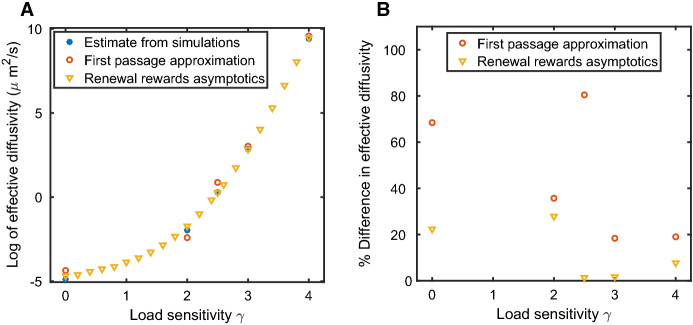
Comparison of effective diffusivity estimates for parallel microtubules driven bidirectionally by kinesin motors as a function of the scaled load sensitivity $\gamma$. **a** Stochastic simulations from Allard et al. (2019) are marked with blue stars, first passage time approximation in Allard et al. (2019) marked with red circles, and renewal reward calculation marked with yellow triangles. Following Allard et al. (2019), we only allow states with *i* kinesin motors moving forward and $K-i$ kinesin motors moving backward, with $0\le i \le K$ and $K=35$ (see Table 3 in “Appendix” for all parameters). The vertical axis is plotted on a log scale to allow for visualization of differences between effective diffusivity estimated from simulations and analytical approximations. **b** Percent error of the first passage approximation in red circles and of the renewal reward calculation in yellow triangles with respect to the simulation results in Allard et al. (2019)

### Microtubule Sliding Model

As a final example of the applicability of our method, we consider a recent investigation into microtubule motility and sliding by Allard et al. (2019). The authors consider a continuous-time Markov chain model of the interaction of two parallel microtubules, cross-linked and moved by multiple identical kinesin motors. Depending on which microtubule the motor heads are attached to, they push the microtubule pair apart in one of the two directions (one of which is arbitrarily assigned to be “positive”). The model assumes that motor attachment to microtubules occurs quickly relative to detachment, allowing a reduced number of dynamic states with microtubules driven by *i* motors pushing in the positive direction and $K-i$ motors pushing in the negative direction (where *K* is the maximal number of motors that fit the overlap region between the two parallel microtubules). The detachment rates are therefore given by

$$\begin{aligned} \kappa _i^+&= (K-i)\kappa _0 \exp {\left( \gamma \frac{i}{K}\right) } \,, \nonumber \\ \kappa _i^-&= i\kappa _0 \exp {\left( \gamma \frac{K-i}{K}\right) } \,, \end{aligned}$$

where $\kappa _i^+$ is the rate at which a motor pulling in the negative direction is replaced by one pulling in the positive direction, $\kappa _i^-$ is the is the rate at which a motor pulling in the positive direction is replaced by one moving in the negative direction, $\kappa _0$ is a force-free transition rate, and $\gamma$ is a dimensionless load sensitivity defined as twice the stall force divided by the detachment force scale (Allard et al. 2019). The relative velocity of the parallel microtubules in each state is given by

$$\begin{aligned} \varDelta v_i&= V_m \frac{2i-K}{K} \,, \end{aligned}$$

where $V_m$ is the speed of a single motor (Allard et al. 2019).

A main point of this study is that parallel microtubules may slide bidirectionally with respect to each other, with a zero mean velocity due to symmetry; thus, the long-term microtubule transport is characterized by diffusive behavior. The effective diffusivity of the microtubule pair driven by a total of 35 motors is measured in Allard et al. (2019) through fitting the slope of the mean squared displacement in stochastic simulations at long time (stars in Fig. 6a) and comparing to a theoretical approximation in terms of a first passage time problem (open circles in Fig. 6a).

Our method based on renewal reward theory yields predictions of the effective diffusivity that are closer to the estimates derived from large-time stochastic simulations (marked with triangles in Fig. 6a). This is further illustrated in Fig. 6b, which shows the percentage error of the approximations with respect to the available simulation estimates in Allard et al. (2019). To make the relationship between effective diffusivity and load sensitivity more clear, we illustrate results for many intermediary values. Our proposed analytical framework also facilitates the possibility of subsequent systematic asymptotic approximations to study dependence on underlying biophysical parameters.

## Discussion

In this work, we consider examples from the intracellular transport literature where particles undergo switching dynamics. In particular, we are interested in determining the effective velocity and diffusivity as well as the expected run length of these particles as they switch between biophysical behaviors such as diffusion, active transport, and stationary states. We propose a method that is based on defining the underlying Markov chain of state switches and the independent cycles of the dynamics marked by returns to a chosen base state. Emphasizing the cyclic structure of the behavior allows us to treat the time durations and spatial displacements of particles in these regenerative cycles as the cycle durations and rewards in a renewal reward process. Through calculation of the statistics of cycle time and displacement, this robust framework provides a rigorous means to study how the asymptotic behavior of switching systems depends on model parameters.

We have restricted our considerations to the case where the switching dynamics and transport coefficients within each state have no spatial dependence. We expect, just as in QSS and homogenization methods, that the regenerative approach will also apply when the dependence between the switching and transport behavior is slowly varying in space, giving now effective velocity and diffusivity that depend on space over the same large scale. In QSS, this requires a scale separation in which switching kinetics are fast relative to transport over spatial variations; see, for example, Newby and Bressloff (2010a); Bressloff and Newby (2013). Homogenization will also work under these conditions, as well as to a broader class of models where the transport coefficients within each state depend also on smaller spatial scales, where its results would depart from those of QSS. Both of these PDE-based approaches produce effective Fokker–Planck equations that give unambiguous interpretation of spatially dependent diffusivity in the context of the equivalent stochastic description.

We expect the regenerative cycle approach developed here should also apply in a generalization of the context in which QSS works, namely, when the spatial scale of transport over a regeneration cycle is small compared to the spatial variation in the switching kinetics and/or transport coefficients within each state. The sense in which to interpret the resulting spatially dependent diffusivity would presumably be the same as for QSS, but this would require a detailed derivation to investigate. The extension of our approach to compute effective transport when the switching and/or state-dependent transport coefficients depend on small as well as large spatial scales is more challenging and problematic because regeneration of the stochastic process would require not only a return to the base state but also to the same values of the transport coefficients. Similarly, while the presence of spatial boundaries can be handled in a QSS framework via a boundary layer analysis of the forward Kolmogorov equation (Zmurchok et al. 2017), spatial boundaries would be a rather difficult challenge for the regeneration cycle analysis because the hitting of the boundary during a cycle would at least ostensibly disrupt its generic statistical properties. In particular, our extension to switching dynamics that are not fully Markovian moves the analysis outside of the framework of partial differential equations, so singular perturbation analysis as in QSS and homogenization cannot be so flexibly applied.

We have emphasized the application of the regenerative cycle methodology to effective transport coefficients, particularly for canonical tug-of-war models describing the transport of cargo by teams of molecular motor proteins. Previous investigations of the effective transport of cargo in these multi-state models have considered individual trajectories of the dynamics, computed using Monte Carlo simulations with the Gillespie algorithm (Müller et al. 2008; Kunwar and Mogilner 2010; Müller et al. 2010). These studies determine the effective velocity of the particles analytically by calculating the distribution of the number of bound motors from the stationary solution of the master equation (Klumpp and Lipowsky 2005). However, determining the effective diffusivity in these studies relied on numerical simulations. Our method proposes a faster and explicit investigation of the impact of model parameters on the effective diffusivity. For instance, Fig. 4 (top right) captures the different behavior of identical motor teams involved in tug-of-war dynamics when the ratio of stall to detachment force is small (weak motors with small effective diffusivity) versus large (strong motors with increasing effective diffusivity). This observation is consistent with simulations in Müller et al. (2008), where the large force ratios correspond to a dynamic instability where only one motor type is primarily bound at the end of an unbinding cascade (Müller et al. 2008, 2010).

Multiple experiments summarized in Hancock (2014) have shown that inhibition of one motor type reduces transport in both directions in several systems, suggesting a “paradox of co-dependence” in bidirectional cargo transport. Several mechanisms accounting for this paradox were proposed, including the microtubule tethering mechanism recently explored in Smith and McKinley (2018). The hypothesis for this mechanism is that motors switch between directed active transport and a weak binding or diffusive state. The recent experimental study in Feng et al. (2018) suggests that teams of kinesin-1 motors coordinate transport using help from the dynamic tethering of kinesin-2 motors. This work shows that when kinesin-1 motors detach, tethering of kinesin-2 to the microtubule ensures that cargo stays near the filament to allow for subsequent reattachment (Feng et al. 2018). Our approach allows us to assess the dependence of the dynamics on a potentially increased reattachment rate for cargo that is already bound to the filament by at least one motor (Fig. 5). Implementing this change in the standard binding models in Müller et al. (2008); Kunwar and Mogilner (2010); Müller et al. (2010) for both kinesin-1/dynein and kinesin-1/DDB dynamics shows a decrease in overall effective velocity, but very large increases in potential run length. This could be consistent with the paradox in that experimentalists would observe more kinesin-directed activity when the reattachment rate is sufficiently high.

We have made MATLAB and Mathematica sample code available for the calculation of effective velocity, diffusivity, and run lengths in a cooperative model of one-directional transport (as discussed in Sect. 5.1) and a tug-of-war model of bidirectional transport (as discussed in Sect. 5.2) (GitHub 2019). The code for these examples can be readily adapted to allow for a general probability transition matrix for the state dynamics, together with the probability distributions for the times and displacement in each state, to extend to other models of the processive movement of molecular motors and cargo transport. As the theory of how motors coordinate to transport cargo continues to develop at a rapid pace, the analysis developed here will provide a tool for new models accounting for tethered and weakly binding states with stochastic transitions whose rates do not depend on spatial position. The framework also extends to complex models with diffusion and binding reactions in higher dimensions, where the moments of times and spatial displacements in each state may be estimated using simulation and the calculation of effective transport quantities reduces to calculation of these moments.

## Appendix

In this appendix, we provide tables with parameter values from the models considered in Sect. 5.

**Table 1:** Parameters for the plus-end motor (conventional kinesin-1) and minus-end motor (cytoplasmic dynein) in Example 4 (Sect. 2.3) and in Sect. 5.2, from Klumpp and Lipowsky (2005), Müller et al. (2008)

Motor	Parameter	Meaning	Value
Plus-end motor	$F_{s+}$	Stall force	6 pN
	$F_{d+}$	Detachment force	3 pN
	$\epsilon _{0+}$	Load-free unbinding rate	1 $\hbox {s}^{-1}$
	$\pi _+$	Binding rate	5 $\hbox {s}^{-1}$
	$v_{f+}$	Forward velocity	1 $\upmu$m/s
	$v_{b+}$	Backward velocity	0.006 $\upmu$m/s
Minus-end motor	$F_{s-}$	Stall force	1.1 pN
	$F_{d-}$	Detachment force	0.75 pN
	$\epsilon _{0-}$	Load-free unbinding rate	0.27 $\hbox {s}^{-1}$
	$\pi _{0-}$	Binding rate	1.6 $\hbox {s}^{-1}$
	$v_{f-}$	Forward velocity	0.65 $\upmu$m/s
	$v_{b-}$	Backward velocity	0.072 $\upmu$m/s

The notation in Example 3 (Sects. 2.3 and  5.1) corresponds to the plus-end motor in this table (kinesin-1)

**Table 2:** Parameters for the DDB complex from Ohashi et al. (2019) in the reattachment model in Sect. 5.3

Parameter	Meaning	Value
$F_s$	Stall force	7 pN
$F_d$	Detachment force	3 pN
$\epsilon _{0-}$	Load-free unbinding rate	0.25 $\hbox {s}^{-1}$
$\pi _{0-}$	Binding rate	1.5 $\hbox {s}^{-1}$
$v_f$	Forward velocity	1 $\upmu$m/s
$v_b$	Backward velocity	0.006 $\upmu$m/s

**Table 3:** Parameters from Allard et al. (2019) for the microtubule sliding model in Sect. 5.4

Parameter	Meaning	Value
*K*	Maximal number of motors	35
$\kappa _0$	Force-less transition rate	0.5 $\hbox {s}^{-1}$
$\gamma$	Dimensionless load sensitivity	3
$V_m$	Single kinesin velocity	0.57 $\upmu$m/s
